# Enhancer-driven Shh signaling promotes glia-to-mesenchyme transition during bone repair

**DOI:** 10.1038/s41413-024-00396-8

**Published:** 2025-01-26

**Authors:** Xin Shen, Hang Zhang, Zesheng Song, Yangjiele Dong, Xiao Ge, Shenghao Jin, Songsong Guo, Ping Zhang, Yu Fu, Yuchi Zhu, Na Xiao, Dongmiao Wang, Jie Cheng, Rongyao Xu, Hongbing Jiang

**Affiliations:** 1 Jiangsu Province Key Laboratory of Oral Diseases, Nanjing, Jiangsu Province China; 2 Department of Oral and Maxillofacial Surgery, The Affiliated Stomatological Hospital of Nanjing Medical University, Nanjing, Jiangsu Province China; 3 Jiangsu Province Engineering Research Center of Stomatological Translational Medicine, Nanjing, Jiangsu Province China

**Keywords:** Bone, Dental diseases

## Abstract

Plp1-lineage Schwann cells (SCs) of peripheral nerve play a critical role in vascular remodeling and osteogenic differentiation during the early stage of bone healing, and the abnormal plasticity of SCs would jeopardize the bone regeneration. However, how Plp1-lineage cells respond to injury and initiate the vascularized osteogenesis remains incompletely understood. Here, by employing single-cell transcriptional profiling combined with lineage-specific tracing models, we uncover that Plp1-lineage cells undergoing injury-induced glia-to-MSCs transition contributed to osteogenesis and revascularization in the initial stage of bone injury. Importantly, our data demonstrated that the Sonic hedgehog (Shh) signaling was responsible for the transition process initiation, which was strongly activated by c-Jun/SIRT6/BAF170 complex-driven *Shh* enhancers. Collectively, these findings depict an injury-specific niche signal-mediated Plp1-lineage cells transition towards Gli1^+^ MSCs and may be instructive for approaches to promote bone regeneration during aging or other bone diseases.

## Introduction

The healing of craniofacial bone injuries poses significant challenges due to unique anatomical and physiological characteristics, compounded by diminished regenerative capacities in aging or diseased bones.^1–4^ Injury-induced bone regeneration entails complex interactions between the neurovascular network and osteo-lineage cells (OLCs).^5,6^ Notably, Gli1^+^ cells, a critical subset of mesenchymal stromal cells (MSCs), play a pivotal role in supporting bone formation within the injury microenvironment and promote the formation of specialized CD31^+^EMCN^+^ type H vessels essential for regenerative repair.^7–11^ Schwann cells (SCs), the essential glial cells in peripheral nerves as marked by Plp1, one of the structural myelin constituents, may serve as key regulators in neurovascular network-associated bone repair. Although there is growing evidence of SCs’ role in tissue repair,^12–16^ the mechanisms by which Plp1-lineage cells fate transitions influence osteogenesis and revascularization remain poorly understood.

It is widely accepted that adult tissue-specific MSCs are established during the embryonic period.^17^ Given that both skeletal stem cells and peripheral nerves in craniofacial bone derive from the neural crest, Plp1^+^ SCs and their precursors likely represent a subpopulation within this adult MSC reservoir, contributing to bone regeneration.^18^ A key open question is whether it can be modulated by de novo generation of MSCs in response to tissue demand. Notably, nerve damage occurs in nearly all types of tissue injuries, and bone healing is increasingly recognized as a nerve-related process.^19,20^ SCs, which serve as axonal sheaths or envelop nerve fibers in Remak bundles, are among the first cells to reappear at injury sites. They display a remarkable ability to reprogram and revert to a progenitor-like state.^13,21,22^ Ex vivo, Plp1^+^ SCs can undergo a transdifferentiation process known as glia-to-MSC transition (GMT),^23^ suggesting that Plp1-lineage cells may have broader implications in bone repair than previously recognized.

Before SCs transition, these cells exhibit a loss of myelin differentiation and activate a series of signaling pathways that support regeneration.^15^ The Sonic hedgehog (Shh), a principal ligand of the Hedgehog (Hh) pathway secreted by neurovascular bundles (NVBs), is suggested to regulate the expression of Gli1 and play a pivotal role in initiating SCs transition.^24–27^ It has been shown that the transcription of the *Shh* gene can be activated by the pioneer transcription factor c-Jun, which binds to injury-induced enhancers of *Shh* in SCs.^28,29^ Recent research has demonstrated that in addition to its role in regulating bone homeostasis during aging through the deacetylation of H3K9ac and H3K56ac,^30^ SIRT6 can also recruit the BRG/BRM-associated factor (BAF) chromatin remodeling subunit BAF170 to gene enhancer regions via its ADP-ribosylation activity, thereby facilitating rapid gene transcription.^31^ However, it is still unclear whether SIRT6 is directly involved in initiating the GMT process through enhancer-driven Shh signaling, or if reduced expression of SIRT6 in SCs is linked to age-related delays in bone repair.

In this study, utilizing multiple lineage tracing strategies and single-cell RNA sequencing (scRNA-seq) assays, we first revealed that the Shh signaling induced by injury signals, responsible for the Plp1-lineage cells to Gli1^+^ MSCs transition, was driven by enhancer-mediated Shh transcription, which then coordinated osteogenesis and revascularization during bone repair. Mechanistically, a c-Jun/SIRT6/BAF170 complex was found to activate Shh transcription by directly binding to injury-induced enhancers. Lastly, we further confirmed the mechanism of Plp1-lineage cells transition in bone repair using a lineage-specific SIRT6 knockout mouse model and human mandibular callus samples. These findings provide a comprehensive understanding of the heterogeneity of MSCs and the critical role of Plp1-lineage cells fate in regulating tissue homeostasis and regeneration.

## Results

### Mandibular denervation impairs the healing of bone injury

**Fig. 1:**
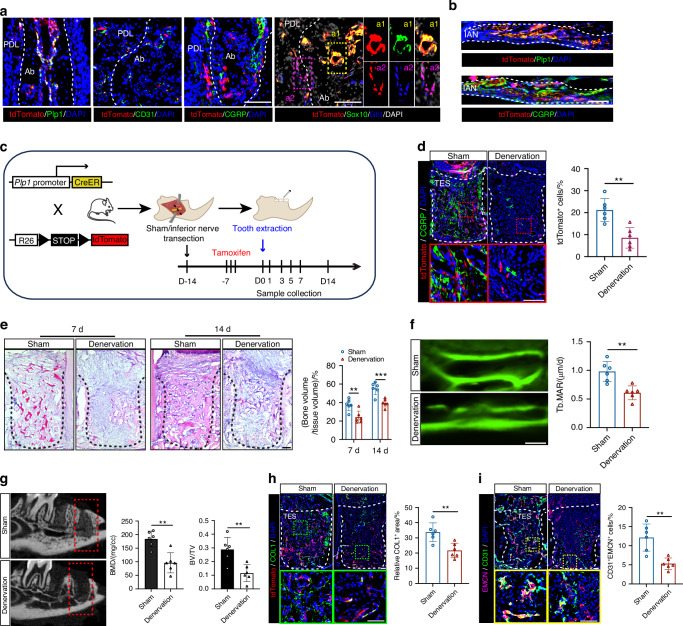
Proximal mandibular denervation impairs the healing of bone injury. **a** Representative images of Plp1, CD31, CGRP Sox10, and Gli1 immunostaining and tdTomato^+^ cells to evaluate the efficiency and specificity of recombination of the *Plp1-creER*^*T2*^ alleles. Scale bar: 100 μm. Ab alveolar bone, PDL periodontal ligament. **b** Representative images of Plp1 and CGRP immunostaining and tdTomato^+^ cells in IAN. Scale bar: 100 μm. IAN inferior alveolar nerve. **c** Schematic illustration of the lineage tracing approach and proximal mandibular denervation in the jaw bone healing model. **d** Representative images of CGRP immunostaining and tdTomato^+^ cells in healing sockets of Sham and Denervation mice at day 7 post tooth extraction and quantification of tdTomato^+^ cells in TES (*n* = 6). Dotted lines indicate the tooth sockets. TES: tooth extraction socket. Scale bar: 100 μm. **e** *H&E* staining of tooth sockets from Sham and Denervation mice at day 7 and day 14 post tooth extraction (*n* = 6). Dotted lines indicate the tooth sockets. Scale bar: 100 μm. **f** Dynamic histomorphometry of trabecular bone (Tb) with quantification of mineralization apposition rate (MAR) in tooth extracted sockets (*n* = 6). Sham and Denervation mice were injected with calcein 7 and 2 days before sacrifice, respectively. Scale bar: 5 μm. **g** Representative images of μCT reconstruction of the alveolar bone regeneration at day 7 post tooth extraction and quantitative analyses on bone volume/total volume (BV/TV) and bone mineral density (BMD) (*n* = 6). **h** Representative images of COL1 immunostaining and tdTomato^+^ cells and quantification of COL1^+^ areas per socket (*n* = 6). Scale bar: 100 μm. **i** Representative images of CD31 and EMCN immunostaining and quantification of CD31^+^EMCN^+^ type H vessels in TES (*n* = 6). Scale bar: 100 μm. Data were presented as mean ± SD; **P < 0.01, ***P < 0.001

To explore the response of Plp1-lineage cells to bone injury, we utilized the tooth extraction socket (TES) model in *Plp1-creER*^*T2*^; *tdTomato* reporter mice, where *Plp1*^+^ cells were marked with tdTomato fluorescence. In the non-injury regions of alveolar bones and inferior alveolar nerve (IAN) (Fig. 1b), tdTomato^+^ cells stained positive for SCs markers (Plp1 and Sox10) but not for endothelial cell marker CD31 or sensory neuron marker CGRP, confirming the specificity and efficiency of our lineage tracing (Fig. 1a). Noticeably, we also found that a small population of tdTomato^+^Sox10^−^ cells were Gli1 positive (Fig. 1a, a2). With the increased bone formation and maturation from day 1 to day 28, tdTomato^+^ cells keep expanding from periodontal ligament (PDL) into the sockets and the quantification of cells density supported these observations (*n* = 3 for each time point) (Fig. S1A, B). However, no obvious tdTomato signal was observed in the healing TES from NC group (*Plp1-creER*^*T2*^*; tdTomato* mice without tamoxifen pre-treatment) (Fig. S1A).

To assess the impact of *Plp1*^*+*^ SCs invasion on bone healing, we employed a classical model of surgical denervation.^21^ TES model is a classic model for studying mandibular injury repair. Given the rich periosteal nerves and *Plp1*^*+*^ SCs distributed in the alveolar bone and the PDL surrounding the root, tooth extractions were performed with or without ipsilateral IAN transection (Fig. 1c). Mechanical hypoalgesia in denervated mice was confirmed through von Frey testing (*P* < 0.01, *n* = 6) (Fig. S1E) and the images of bone trabecula (Fig. S1C) and IAN (Fig. S1F) in mandible illustrated the degeneration of tdTomato^+^ cells in denervated mice (*P* < 0.001, *n* = 6). CD31 immunostaining results in non-injury regions revealed no significant differences, ensuring the removal of the nerve supply does not impact blood supply within mandibles (*P* > 0.05, *n* = 6) (Fig. S1D). The denervation significantly reduced tdTomato^+^ cell density (Fig. 1d) and bone volume at both day 7 and day 14 post-extraction, as shown by micro-CT scans and histological analysis (*P* < 0.01, *n* = 6) (Figs. 1e, g and S1G). Delayed bone formation in denervated mice was further evidenced by double-labeling analysis (*P* < 0.01, *n* = 6) (Fig. 1f). At day 7 post-injury, a reduced expression of type 1 collagen (COL1) was observed in the denervation group (*P* < 0.01, *n* = 6), with a subset also being tdTomato positive (Fig. 1h). Additionally, denervated mice displayed a lower density of type H vessels (*P* < 0.01, *n* = 6), characterized by high co-expression of CD31 and Endomucin (EMCN) (Fig. 1i). These findings collectively underscore the critical regulatory role of peripheral innervation in bone healing.

### Identification of oeteoprogenitor cells with glial characteristics by scRNA-seq during bone repair

**Fig. 2:**
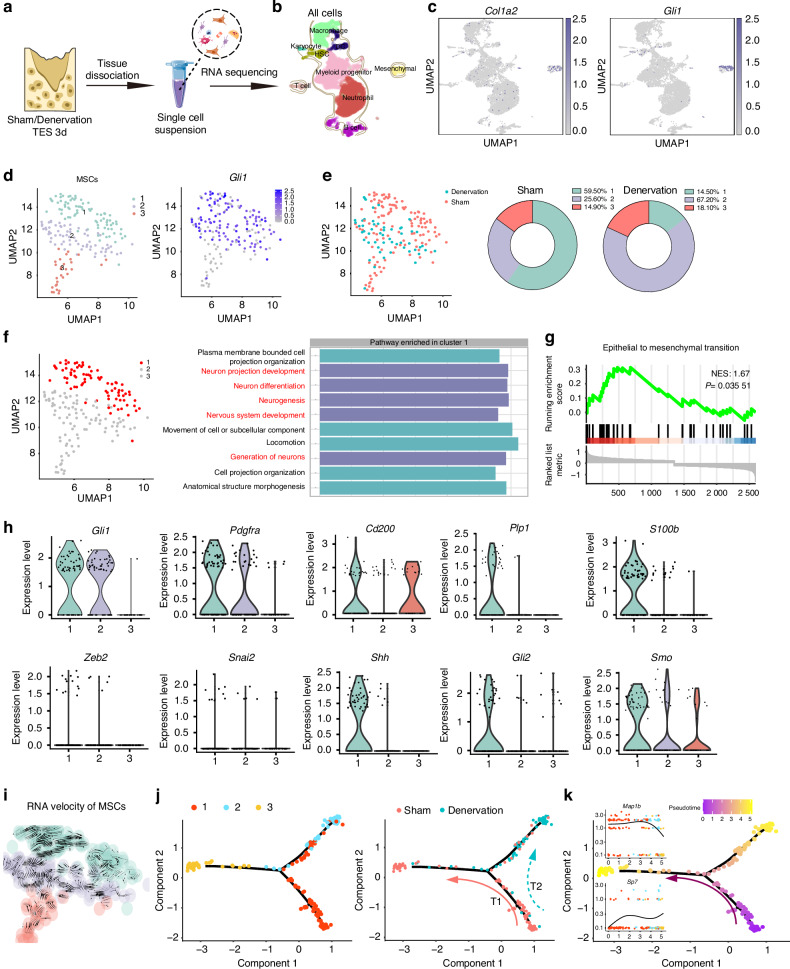
Identification of oeteoprogenitor cells with glial characteristics by scRNA-seq during bone repair. **a** Flow chart of preparation of scRNA-seq samples from Sham and Denervation mice tooth extraction sockets. **b** Cell identities obtained through scRNA-seq were visualized via Uniform Manifold Approximation and Projection (UMAP). Various cell populations were demarcated and distinguished by color. Each point represented an independent cell. **c** The expression levels of stromal cell marker genes *Col1a2* and *Gli1* were projected onto UMAP atlas. **d** Subclustering of stromal cells reveals three cell clusters. Subclusters 1 and 2 express MSC marker *Gli1*. **e** The distribution of sham and denervation samples across stromal subcluster. **f** GO enrichment analysis of the biological functions of subcluster 1. **g** GSEA analysis indicating activation of a mesenchymal transition program of subcluster 1. **h** Violin plots showing increased co-expression of mesenchymal, neural glial cell markers, mesenchymal transition drivers, and Shh signaling associated markers in subcluster 1. **i** RNA velocities projected onto UMAP embedding of stromal cells subclusters. **j**, **k** Pseudotime lineage trajectory analysis of stromal cells subclusters. Inset: decreasing expression of glial marker *Map1b* and increasing expression of osteogenetic marker *Sp7*. T1: trajectory 1; T2: trajectory 2

To elucidate the regulatory role of peripheral innervation in bone repair, we conducted scRNA-seq on both sham and denervated TES at day 3 post-extraction, a critical time point for the initiation of regeneration activities (Fig. 2a). A total number of 7 707 cells were obtained with 17 441 genes of each cell. We preprocessed the dataset with Seurat package. Only cells exhibiting between 200 and 6 000 expressed genes, and a mitochondrial unique molecular identifier (UMI) rate of less than 20%, were retained post cell-quality filtration (Fig. S2A). After quality control, 2 000 genes with the most variable value from 17 441 genes were selected for subsequent analysis. The UMAP dimensional reduction method identified nine distinct clusters (Fig. 2b), each characterized by unique cell markers including macrophages (*Ccr2* and *Csf1r*), dendritic cells (*Siglech*), karyocytes (*Ms4a2*), hematopoietic stem and progenitor cells (*Cd34*, *Kit*, and *Ly6a*), myeloid progenitors (*Mpo*), B cells (*Cd79a*), T cells (*Cd3e*), neutrophils (*Csf3r*), and MSCs marked by *Col1a2* and *Gli1* (Figs. 2c and S2C). The top 5 expressed genes expressed in each defined cell type were identified and compared (Fig. S2B). Despite non-immune cells (MSCs) accounting for only 2.20% of all identified cells, we prioritized them due to their critical role in initiating bone regeneration and these MSCs could be divided into three subclusters (Fig. 2d). Cluster 3 was identified as OLCs for expression of *Bmp2* and *Bglap* and enrichment in genes with established roles in osteoblast differentiation (Fig. S2E, F). Clusters 1 and 2 were identified as Gli1^+^ MSCs due to their high expression of *Gli1* (Fig. 2d). Cluster 2, in particular, exhibited characteristics such as negative regulation of osteoblast differentiation and the capacity of cell migration, among others (Fig. S2D). Interestingly, while cluster 2 was equally represented in both sham and denervation groups, cluster 1 was enriched in sham TES and almost disappeared in denervation TES (Fig. 2e), indicating its relationship with inferior nerve and the healing of bone injury.

To explore potential role of cluster 1, we performed Gene Ontology analysis and found several nervous system-related pathways were activated (Fig. 2f). Violin plots analysis consistently identified cluster 1 as a distinct subset of Gli1^+^ MSCs that was characterized by co-expression of mesenchymal transcripts (*Gli1*, *Pdgfra*), osteo-lineage cells marker (*Cd200*), neural glial markers (*Plp1* and *S100b*), and transcription factors involved in epithelial-to-mesenchymal transition (EMT) regulation (*Snai2* and *Zeb2*), opening the possibility of the existence of a “transitional” population expressing neural glial and mesenchymal transcripts indicative of the transition process (Fig. 2h). This notion was corroborated by striking enrichment of gene sets reflecting EMT (Fig. 2g, normalized enrichment score, 1.67; *P* = 0.035 51). To further investigate the transcriptional dynamics of MSCs differentiation, RNA velocity analysis was performed which predicts the future states of cellular subpopulations by taking into account the relative abundance of both nascent (unspliced) and mature (spliced) mRNA. Our findings revealed a differentiation trajectory originating from cluster 1, which was identified to be located in the upstream of the differentiation trajectory of OLCs (cluster 3), matching observations from above analyses (Fig. 2i). The experimental grouping information was projected onto the pseudotime trajectory, showing that cluster 1 has two potential differentiation trajectories. During normal bone healing, cluster 1 may differentiate into OLCs subcluster via trajectory 1 (T1) (Fig. 2j, k), with a stepwise decrease in expression of SCs marker *Map1b* and increase in expression of osteogenic marker *Sp7* during the pseudotime (Fig. 2k, inset). However, it may switch to trajectory 2 (T2) and differentiate into cluster 2 after denervation, indicating the loss of osteogenic differentiation capacity. Collectively, these findings identified a subcluster of Gli1^+^ oeteoprogenitor cells with glial signature at the transcriptional level, which was computationally predicted to give rise to the formation of osteogenic niche during the healing of bone injury.

### Plp1-lineage cells contribute to Gli1^+^ MSCs formation and promote bone regeneration

**Fig. 3:**
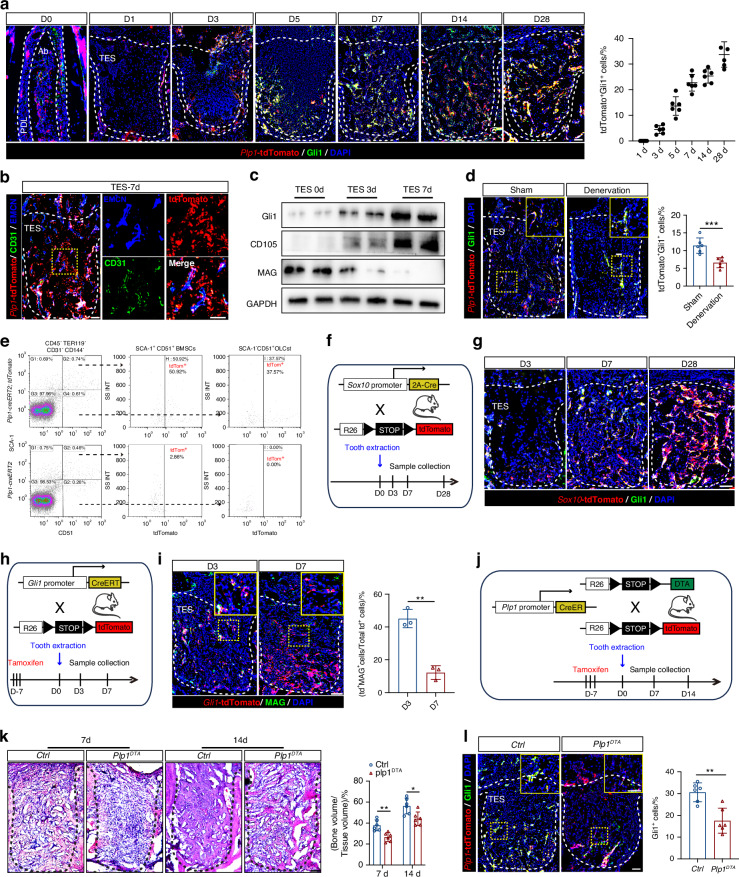
Plp1-lineage cells contribute to Gli1^+^ MSCs formation and promote bone regeneration. **a** Representative images of tdTomato^+^ cells and Gli1 immunostaining during jaw bone regeneration and quantification of tdTomato^+^ Gli1^+^ cells in TES from *Plp1-creER*^*T2*^*; tdTomato* mice (*n* = 6). Scale bar: 100 μm. **b** Representative images of tdTomato^+^ cells combined with CD31 and EMCN immunostaining at day 7 post tooth extraction. **c** Western blot images of MAG, CD105, Gli1 expression of FACS-sorted tdTomato^+^ cells from tooth extraction sockets at day 0, 3, and 7 post tooth extraction. **d** Representative co-localization images of tdTomato^+^ cells and Gli1^+^ cells in healing sockets of Sham and Denervation mice at day 7 post tooth extraction and relative quantification (*n* = 6). Scale bar: 100 μm. **e** Representative flow cytometry plots depicting the gating strategy to identify MSCs and OLCs in TES of *Plp1-creER*^*T2*^*; tdTomato* and *Plp1-creER*^*T2*^ mice 7 days post tooth extraction. **f** Schematic illustration of the lineage tracing approach in the jaw bone healing model using *Sox10-cre; tdTomato* mice. **g** Representative images of tdTomato^+^ cells and Gli1 immunostaining during jaw bone regeneration in TES from *Sox10-cre; tdTomato* mice. Scale bar: 100 μm. **h** Schematic illustration of the lineage tracing approach in the jaw bone healing model using *Gli1-creER*^*T2*^*; tdTomato* mice. **i** Representative images of tdTomato^+^ cells and MAG immunostaining during jaw bone regeneration and quantification of tdTomato^+^ MAG^+^ cells in TES from *Gli1-creER*^*T2*^*; tdTomato* mice (*n* = 3). Scale bar: 100 μm. **j** Schematic of the experimental design to ablate SCs using an inducible diphtheria toxin allele (DTA). **k** *H&E* staining of tooth sockets from control and *Plp1*^*DTA*^ mice at day 7 and day 14 post tooth extraction (*n* = 6). Scale bar: 100 μm. **l** Representative images of tdTomato^+^ cells and Gli1 immunostaining in TES of control and *Plp1*^*DTA*^ mice at day 7 post tooth extraction and relative quantification per socket (*n* = 6). Scale bar: 100 μm. Data were presented as mean ± SD; *P < 0.05, **P < 0.01, ***P < 0.001

To define experimentally if Plp1-lineage cells contribute to the formation of Gli1^+^ MSCs via GMT process, we analyzed TES sections from *Plp1-creER*^*T2*^*; tdTomato* mice co-stained for Gli1. We observed a progressive increase in MSCs of *Plp1* origin from day 0 to day 28 post-injury, signifying co-expression of tdTomato and Gli1(*n* = 6 for each time point). Before tooth extraction, tdTomato^+^ cells were detected in PDL and alveolar bone marrow, mostly being negative for Gli1 expression. From day 1 to day 28, tdTomato^+^ cells began to migrate from PDL into the sockets and keep expanding with the percentage of tdTomato^+^Gli1^+^ rising to (35.8 ± 7.9)% at day 28 (Fig. 3a), accompanied by the formation of type H vessels (Fig. 3b). Other mesenchymal markers CD105 and NG2 were also found to be co-expressed with tdTomato in TES (Fig. S3E). In addition, increasing tdTomato^+^ cells in TES were also positive for osteogenic marker osteopontin (OPN) at day 14 and day 28 (*P* < 0.001, *n* = 6), indicating the differentiation from *Plp1*-tdTomato^+^ cells to OLCs during the maturation stage of TES healing (Fig. S3A). Furthermore, *Plp1*-tdTomato^+^ cells in TES from 0 (alveolar bone), 3, 7 days after tooth extraction sorted by FACS revealed that the expression of the mature myelination marker MAG in SCs gradually decreased, while the expression of MSCs markers CD105, and Gli1 progressively increased (*P* < 0.05, *n* = 3) (Figs. 3c and S3C). RT-qPCR further confirmed this trend with a decline in *Mag* mRNA and an upregulation of *Eng* and *Gli1* (*P* < 0.05, *n* = 3) (Fig. S3D). Notably, the presence of tdTomato^+^Gli1^+^ cells was significantly reduced in denervated TES at day 7 (*P* < 0.001, *n* = 6) (Fig. 3d). Compared to the *Plp1-creER*^*T2*^ negative littermate control mice, a significant proportion (50.92 ± 1.49%) of highly purified alveolar bone MSCs from day 7 TES, defined by CD45^−^Ter119^−^CD31^−^CD144^−^Sca1^+^CD51^+^ expression was found to be of glial origin as indicated by tdTomato^+^ in *Plp1-creER*^*T2*^; *tdTomato* mice. However, lower proportion [(37.57 ± 5.41)%] of downstream OLCs (CD45^−^Ter119^−^CD31^−^CD144^−^Sca1^−^CD51^+^) were also derived from *Plp1*-tdTomato^+^ cells (Figs. 3e and S3F, G), indicating a great portion (about 62.43%) of OLCs may be from other origins (*n* = 3). The immunostaining results showed that tdTomato^+^ cells were also associated with Runx2-labeled osteoprogenitors (Blue) in the healing region, suggesting its synergistic participation in the early osteogenesis process (Fig. S3B).

Furthermore, another SCs lineage tracing model *Sox10-cre; tdTomato* mice was used to add greater support to the GMT process during the healing of bone injury, showing the increasing co-expression of *Sox10*-tdTomato with Gli1 from d3 to d28 (*n* = 3 for each time point) (Figs. 3f, g and S3I). We also used *Sox10-cre; tdTomato* mice to trace the lineage differentiation and distribution of *Sox10*-positive cells in the development of the mandible. We observed that at postnatal day 3 (P3), *Sox10*^*+*^ cells were distributed around the tooth germ. As the jawbone and teeth developed, *Sox10*^*+*^ cells were widely distributed in pulp, PDL, and alveolar bone marrow space at P60, suggesting their potential involvement in both tooth and bone development (Fig. S3H). *Gli1-creER*^*T2*^*; tdTomato* mice were then used to detect potential multiple populations and the results showed that the number of tdTomato^+^MAG^+^ cells decreased during the healing of bone injury, providing additional evidence for the presence of GMT from a different perspective (*P* < 0.01, *n* = 3) (Fig. 3h, i).

To further substantiate the lineage tracing findings, we generated *Plp1*^*DTA*^ mice and induced the selective ablation of *Plp1*^+^ cells followed by tooth extraction (Fig. 3j). Alveolar bone regions of tamoxifen-treated mice, harvested 3 days post-induction, exhibited widespread cell death among the *Plp1*^+^ cells population (Fig. S3L). von Frey filament stimulus testing showing no obvious mechanical hypoalgesia in *Plp1*^*DTA*^ mice (*P* > 0.05, *n* = 5) (Fig. S3O) and the CGRP^+^ nerve fibers retained in TES after the deletion of SCs (*P* > 0.05, *n* = 6) (Fig. S3P). The volume of bone regenerated was severely suppressed in *Plp1*^*DTA*^ mice at both day 7 and day 14 post-extraction (*P* < 0.01, *n* = 6) (Fig. S3M). Double-labeling analysis revealed defects in new bone formation rate in *Plp1*^*DTA*^ mice by measuring the distance between the 2 fluorescent labels (*P* < 0.01, *n* = 6) (Fig. S3N). Further, markedly reduced Shh and HIF-1α expression were observed after selective ablation of *Plp1*^*+*^ cells (*P* < 0.01, *n* = 6) (Fig. S3Q, R). Histological analysis also revealed a reduced bone volume in TES of *Plp1*^*DTA*^ mice at both day 7 (*P* < 0.01, *n* = 6) and day 14 (*P* < 0.05, *n* = 6) post-extraction (Fig. 3k). Of note, the number of total Gli1^+^ cells remarkably decreased after ablation of *Plp1*^*+*^ cells (*P* < 0.01, *n* = 6) (Fig. 3l). Likewise, the COL1^+^ bone area (*P* < 0.001, *n* = 6) and the density of type H vessels (*P* < 0.01, *n* = 6) also reduced in the TES of *Plp1*^*DTA*^ mice (Fig. S3J, K). Collectively, these data corroborate our finding of a subset of Gli1^+^ MSCs within the TES, which originate from Plp1-lineage cells and play a pivotal role in bone regeneration.

### Hh signaling driven Plp1-lineage cells transition is required for the healing of bone injury

**Fig. 4:**
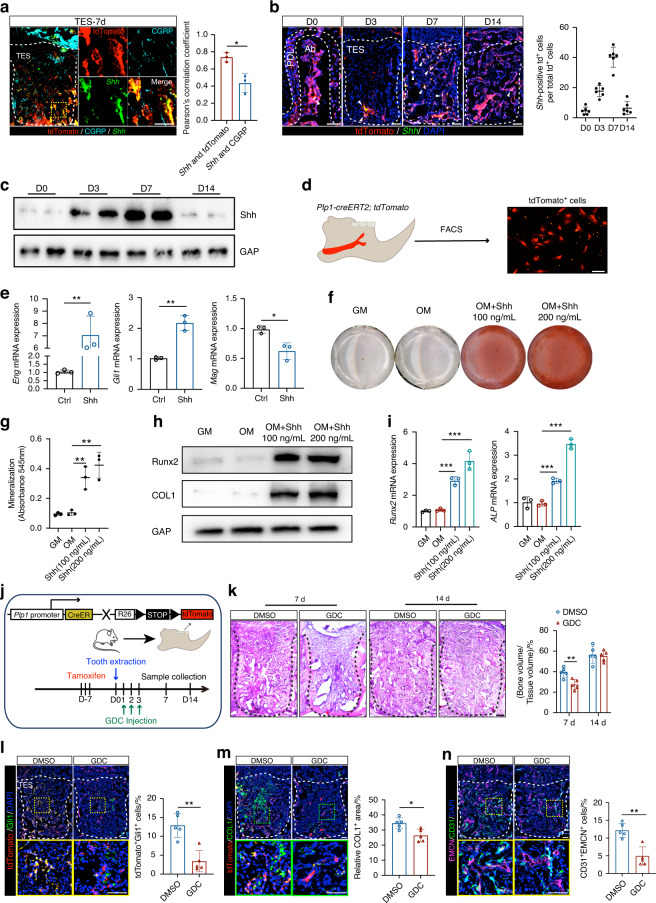
Hh signaling driven Plp1-lineage cells transition is required for the healing of bone injury. **a** Representative images of tdTomato^+^ cells, CGRP immunostaining in conjunction with in situ hybridization of *Shh* mRNA in TES from *Plp1-creER*^*T2*^*; tdTomato* mice. The Pearson’s correlation coefficient is presented as the quantification of *Shh* mRNA signals that were colocalized with tdTomato or CGRP (*n* = 3). Scale bar: 100 μm. **b** Representative images of tdTomato^+^ cells in conjunction with in situ hybridization of *Shh* mRNA and quantification of tdTomato^+^ *Shh*^+^ cells in TES from *Plp1-creER*^*T2*^*; tdTomato* mice (*n* = 6). Scale bar: 100 μm. **c** Western blot images of Shh expression of FACS-sorted tdTomato^+^ cells from tooth extraction sockets at day 0, 3, and 7 post tooth extraction. **d** Schematic illustration of FACS assay and purified tdTomato^+^ cells from mandible. Scale bar: 20 μm. **e** RT-qPCR data of *Eng*, *Gli1*, and *Mag* mRNA expression of SCs treated with recombinant Shh (*n* = 3). **f** Shh converts SCs into osteolineage cells as demonstrated by alizarin red staining (ARS) of FACS-sorted tdTomato^+^ cells cultured in regular growth medium (GM) or osteogenic medium (OM). **g** The quantification of ARS in (**f**) (*n* = 3). **h** Western blot images of Runx2 and COL1 expression of FACS-sorted tdTomato^+^ cells cultured in GM or OM and treated with recombinant Shh. **i** RT-qPCR data of *Runx2* and *Alp* mRNA expression of SCs cultured in GM or OM and treated with recombinant Shh (*n* = 3). **j** Experimental strategy for Hh signaling inhibition in *Plp1-creER*^*T2*^*; tdTomato* mice. **k** *H&E* staining of tooth sockets from DMSO and GDC mice at day 7 and day 14 post tooth extraction and the quantification analysis (*n* = 5). Scale bar: 100 μm. **l**, **m** Representative images of tdTomato^+^ cells and Gli1/COL1 immunostaining in TES at day 7 post tooth extraction and the relative quantification (*n* = 5). Scale bar: 100 μm. **n** Representative images of CD31 and EMCN immunostaining and quantification of CD31^+^EMCN^+^ type H vessels per socket (*n* = 5). Scale bar: 100 μm. Data were presented as mean ± SD; *P < 0.05, **P < 0.01, ***P < 0.001

Building upon the evidence that Plp1-lineage cells give rise to Gli1^+^ MSCs, we explored the mechanisms underlying GMT during bone healing. Initially, our scRNA-seq analysis highlighted the specific expression of *Shh* and downstream Hh signaling effectors, including *Gli1*, *Gli2*, and *Smo*, within cluster 1 (Fig. 2h). First, we detected the *Shh* mRNA expression in TES using FISH staining and found more co-expression of *Shh* with tdTomato than with CGRP, indicating that Shh is more likely secreted from tdTomato^+^ cells rather than neural fibers (*P* < 0.05, *n* = 3) (Fig. 4a). Shh expression was either co-localized with or in close proximity to tdTomato signals (*n* = 6 for each time point) (Fig. S4A, B), suggesting that injury-activated SCs secrete Shh to initiate their GMT. The *Shh* mRNA expression pattern also supports this conclusion (*n* = 6 for each time point) (Fig. 4b). Notably, Shh, recognized as a primary initiator of Gli1 expression and secreted by NVBs within the mandible, exhibited expression patterns that closely paralleled those of Gli1, showing a gradual increase from day 3 to day 7 post-tooth extraction before returning to baseline by day 14 (Fig. 4c). To directly test the role of Shh in driving GMT, we isolated primary tdTomato^+^ cells (SCs) from jaw bones sorted by FACS (Fig. 4d) and treated them with recombinant Shh (100 ng/mL). As expected, Shh significantly suppressed the expression of the SCs marker *Mag* and upregulated the mesenchymal markers *Eng* and *Gli1* (*P* < 0.05, *n* = 3) (Fig. 4e).

Furthermore, to assess whether Plp1-lineage cells not only exhibit phenotypic changes indicative of GMT but also acquire cellular characteristics resembling stromal cell fates, we performed assays to evaluate the differentiation potential of Shh-treated Plp1-lineage cells into OLCs. The results demonstrated a dose-dependent increase in alizarin red staining, indicating that the addition of Shh initiated the differentiation of *Plp1*-tdTomato^+^ cells into OLCs (*P* < 0.01, *n* = 3) (Fig. 4f, g). WB and qRT-PCR results to test osteogenic markers also confirm this conclusion (*P* < 0.01, *n* = 3) (Fig. 4h, i). It is worth noting that cells treated with osteogenic medium alone were almost unable to form mineralized matrix, suggesting that *Plp1*^+^ cells do not have inherent osteogenic potential, but rather acquire the cellular characteristics resembling stromal cell fates to differentiate into OLCs under the activation of the shh signaling. To further investigate the role of Hh signaling in bone healing and GMT, we administered GDC-0449 (Hh signaling inhibitor) for three consecutive days following tooth extraction (Fig. 4j). The results indicated a decrease in tdTomato^+^Gli1^+^ cells (*P* < 0.01, *n* = 5) and no change in total tdTomato^+^ cells (*P* > 0.05, *n* = 5) (Figs. 4l and S4F). Micro-CT and double-labeling analyses revealed defects in bone regeneration at day 7 (*P* < 0.05, *n* = 5) (Fig. S4C) and new bone formation rate (*P* < 0.05, *n* = 5) (Fig. S4D, E) and the number of tdTomato^+^CD105^+^ or tdTomato^+^NG2^+^ cells also decreased after GDC-0449 treatment (*P* < 0.01, *n* = 5) (Fig. S4G, H), signifying an impaired GMT process. Additionally, there was a reduction in COL1^+^ areas in the GDC-0449 treated group (*P* < 0.05, *n* = 5) (Fig. 4m), and micro-CT analysis along with histological assessments revealed a delayed healing in TES on day 7, although no significant differences were observed by day 14 (*P* > 0.05, *n* = 5), suggesting a temporal effect of the inhibitor on healing (Figs. 4k and S4C). Furthermore, a decrease in CD31^+^EMCN^+^ type H vessels was observed following GDC-0449 administration at day 7 (*P* < 0.01, *n* = 5) (Fig. 4n). These findings collectively demonstrate that Shh signaling is indispensable for the GMT and the subsequent healing process of bone injuries.

### Impaired GMT is associated with age-related healing delay of bone injury

**Fig. 5:**
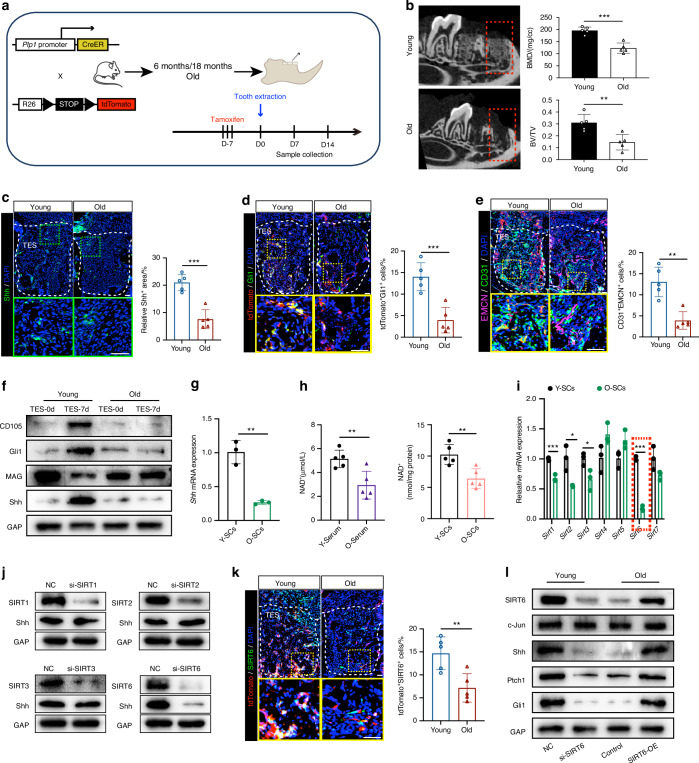
Disrupted GMT of Plp1-lineage cells attenuates bone regeneration in aged mice. **a** Experimental strategy for aged mice lineage tracing and jaw bone healing model. **b** Representative images of μCT reconstruction of the alveolar bone regeneration at day 7 post tooth extraction from young/old mice and quantitative analyses (*n* = 5). **c** Representative images of Shh immunostaining in healing sockets at day 7 post tooth extraction and the quantification (*n* = 5). Scale bar: 100 μm. **d** Representative images of tdTomato^+^ cells and Gli1 immunostaining in healing sockets at day 7 post tooth extraction and relative quantification per socket (*n* = 5). Scale bar: 100 μm. **e** Representative images of CD31 and EMCN immunostaining and quantification of CD31^+^EMCN^+^ type H vessels per socket (*n* = 5). Scale bar: 100 μm. **f** Western blot images of CD105, Gli1, MAG, and Shh expression of FACS-sorted tdTomato^+^ cells from young/old mice tooth extraction sockets at day 0 and 7 post tooth extraction. **g** RT-qPCR data of *Shh* mRNA expression of SCs from young/old mice (*n* = 3). **h** NAD^+^ was measured in serum and supernatant medium of SCs from young and old mice (*n* = 5). Y, young mice. O, old mice. **i** RT-qPCR analysis of mRNA levels of *Sirt1-7* in SCs (*n* = 3). **j** Western blot analysis of the knockdown efficiency of siRNAs for SIRT1, SIRT2, SIRT3, SIRT6, and relevant Shh expression. **k** Representative images of tdTomato^+^ cells and SIRT6 immunostaining in TES at day 7 post tooth extraction and the relative quantification (*n* = 5). Scale bar: 100 μm. **l** Western blot showing SIRT6, c-Jun, Shh, Ptch1, and Gli1 in SCs with aging or SIRT6 knockdown/overexpression. Data were presented as mean ± SD; *P < 0.05, **P < 0.01, ***P < 0.001

Considering the effects of Hh signaling-driven GMT on the healing process of bone injury, we asked whether age-related healing delay was associated with impaired GMT and established the TES model with aged mice (Fig. 5a). Firstly, despite the comparable number of *Plp1*-tdTomato^+^ progenitors in alveolar bones and PDL before bone injury observed in both young (6 months) and old mice (18 months) (*P* > 0.05, *n* = 5) (Fig. S5B), aged mice exhibited a significant reduction in bone volume at day 7 and day 14 post-extraction compared to their younger counterparts (*P* < 0.01, *n* = 5) (Fig. S5A). This delay of bone regeneration was further characterized by decreased BMD and BV/TV in aged mice (*P* < 0.01, *n* = 5) (Figs. 5b and S5C), as well as declined bone formation rate (*P* < 0.01, *n* = 5) (Fig. S5D), reduced areas of COL1^+^ (*P* < 0.05, *n* = 5) (Fig. S5E) and CD31^+^EMCN^+^ type H vessels (Fig. 5e). Additionally, aged mice exhibited markedly lower levels of Shh expression and tdTomato^+^Gli1^+^ cells in the TES at day 7 (*P* < 0.001, *n* = 5) (Fig. 5c, d). Further investigation into SCs from aged mice, isolated via FACS from the alveolar bones (TES-0d) or TES (TES-7d), revealed a diminished ability to initiate GMT as evidenced by nearly invariant expression levels of Shh, the stromal markers CD105, Gli1, and the SCs marker MAG from day 0 to day 7 (Fig. 5f and Fig. S5F). The *Shh* mRNA expression also decreased markedly in SCs from aged mice (*P* < 0.01, *n* = 3) (Fig. 5g). These findings suggest that age-related delays in bone healing may be associated with an impaired GMT of SCs.

To unravel the upstream regulatory mechanisms responsible for age-related Shh downregulation in TES and the compromised GMT, we focused on NAD^+^ metabolism, a known hallmark of aging linked to skeletal diseases.^32^ Both serum and supernatant medium of SCs NAD^+^ levels in aged mice were substantially lower than those in younger mice (*P* < 0.01, *n* = 5) (Fig. 5h). Since sirtuins are NAD^+^-dependent protein deacetylases known to affect bone regeneration,^33^ we investigated their role in regulating Shh expression. Profiling of sirtuin genes in SCs during aging revealed significantly reduced expression of *Sirt1*, *Sirt2*, *Sirt3*, and *Sirt6* (Fig. 5i). Notably, SIRT6 knockdown specifically resulted in a pronounced decrease in Shh expression, an effect not observed with knockdown of other sirtuins (Fig. 5j). Additionally, the reduced expression of SIRT6 in tdTomato^+^ cells further indicated a deficiency of this protein in SCs from aged mice (*P* < 0.01, *n* = 5) (Fig. 5k). To assess the impact of SIRT6 on Hh signaling, we conducted in vitro experiments treating primary SCs from young and old mice with SIRT6 knockdown (si-SIRT6) and overexpression vectors (SIRT6-OE). The results demonstrated that si-SIRT6 significantly reduced the expression of SIRT6, along with notable decreases in Shh, Ptch1, and Gli1 expression in young SCs. Conversely, SIRT6-OE induced the opposite effects in old SCs, enhancing the expression of these markers (Figs. 5l and S5G). Thus, our findings indicate that impaired GMT of SCs contributes to the age-related delay in bone injury healing, potentially attributed to a deficiency in SIRT6.

### c-Jun/SIRT6/BAF170 complex binds to injury-specific enhancers and activates *shh* transcription

**Fig. 6:**
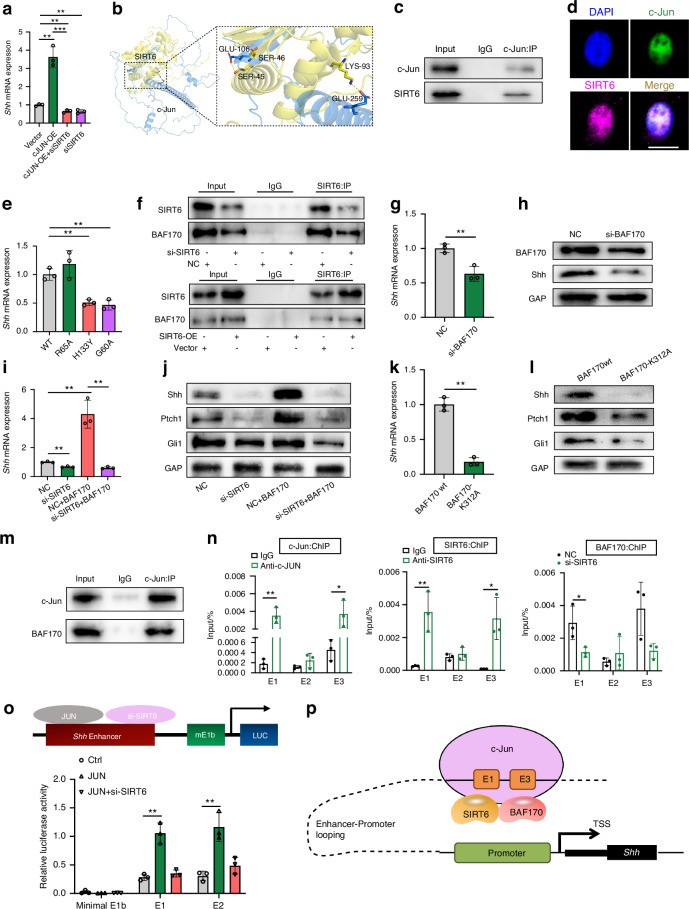
c-Jun/SIRT6/BAF170 complex binds to injury-specific enhancers and activates *shh* transcription in SCs. **a** RT-qPCR analysis of *shh* mRNA level in SCs treated with c-Jun overexpression or SIRT6 knockdown (*n* = 3). **b** 3D modeling of the interaction between c-Jun (blue) and SIRT6 (yellow) proteins elucidated through protein-protein docking predictions from AlphaFold 2. The stick representation delineates the amino acid residues, while yellow dashed lines illustrate the hydrogen bonds. **c** Western blot showing co-immunoprecipitation between SIRT6 and c-Jun. **d** Immunofluorescence images of the colocalization of SIRT6 and c-Jun in SCs. Scale bar: 10 μm. **e** RT-qPCR analysis of *shh* mRNA level in SIRT6 knockdown SCs rescued with different alleles of SIRT6 (*n* = 3). **f** Western blot showing co-immunoprecipitation between SIRT6 and BAF170 with downregulation or upregulation of SIRT6. **g** RT-qPCR analysis of *Shh* mRNA expression after BAF170 knockdown (*n* = 3). **h** Western blot showing the expression of BAF170 and Shh after BAF170 knockdown. **i** RT-qPCR analysis of *shh* mRNA level in SCs treated with SIRT6 knockdown and BAF170 overexpression (*n* = 3). **j** Western blot showing the expression of Shh, Ptch1, and Gli1 in SCs treated with SIRT6 knockdown and BAF170 overexpression. **k** RT-qPCR analysis of *shh* mRNA level in SCs after transfection with a plasmid expressing BAF170-K312A (*n* = 3). **l** Western blot showing the expression of Shh, Ptch1, and Gli1 in SCs treated with BAF170-K312A mutant. **m** Western blot showing co-immunoprecipitation between c-Jun and BAF170. **n** ChIP-qPCR for c-JUN and SIRT6 on three reported injury-specific shh enhancer sites and ChIP-qPCR for BAF170 on shh enhancer sites treated with SIRT6 knockdown (*n* = 3). **o** Luciferase reporter assay for shh enhancer 1 and 3 (*n* = 3). **p** Schematic showing the mechanism that c-Jun/SIRT6/BAF170 complex binds to injury-specific enhancers and activates SCs shh transcription. Data were presented as mean ± SD; *P < 0.05, **P < 0.01, ***P < 0.001

SIRT6, a histone deacetylase known for its role in chromatin condensation and gene suppression,^34^ has been identified as a positive regulator of Shh expression, as demonstrated in our studies (Fig. 5j, l). To elucidate the molecular mechanisms by which SIRT6 influences Shh transcription in SCs, we investigated its potential interacting partners. The c-Jun subunit of the AP-1 transcription factor complex has captured our attention due to its pivotal role in driving SCs’ transcriptional response following injury, controlling the transdifferentiation of myelin and Remak SCs into repair-specific cells.^35^ Notably, c-Jun binds to injury-induced enhancers to activate Shh transcription in SCs involved in repair processes.^25^ Our results showed that overexpression of c-Jun in SCs led to increased *Shh* expression, which was diminished by SIRT6 knockdown (Fig. 6a). Protein–protein docking predictions from AlphaFold 2 indicated that SIRT6 could bind directly to c-Jun through hydrogen bonds (Fig. 6b), and further co-immunoprecipitation (co-IP) assays confirmed the interaction between endogenous SIRT6 and c-Jun (Fig. 6c). Consistently, when Flag-tagged SIRT6 was introduced in HEK293T cells, this ectopic protein was efficiently precipitated by c-Jun antibodies (Fig. S6A). Immunofluorescence studies also validated this interaction (Fig. 6d).

The SIRT6 protein possesses dual enzymatic functionalities: deacetylase and mono-ADP-ribosylase activities.^31^ To discern which of these enzymatic activities of SIRT6 is imperative for the augmentation of Shh transcription, we transfected SCs with functionally distinct SIRT6 mutants: the G60A variant (retaining deacetylase activity but deficient in mono-ADP-ribosylase function) and the R65A variant (competent in mono-ADP-ribosyl transferase activity but lacking deacetylase function). The catalytically dead (H133Y) and G60A alleles of SIRT6 significantly inhibited *Shh* expression while R65A alleles did not compare with WT plasmid, suggesting that ribosylation but not deacetylation activity of SIRT6 is involved in *Shh* transcriptional activation (Fig. 6e). It has been reported that SIRT6 recruits BAF170 to enhancer region of several genes locus and promotes transcriptional enhancement through mono-ADP ribosylation.^31^ We asked whether c-Jun/SIRT6 could recruit BAF170 to *Shh* enhancer regions and promote its mRNA transcription. A co-IP assay in the presence or absence of si-SIRT6 and SIRT6-OE plasmids was performed, verifying the interaction between endogenous SIRT6 and BAF170 (Fig. 6f). Immunofluorescence results also confirmed this conclusion (Fig. S6B). Upon BAF170 knockdown in SCs, Shh expression significantly decreased at both mRNA (*P* < 0.01, *n* = 5) and protein levels (*P* < 0.05, *n* = 3) (Figs. 6g, h and S6E). In contrast, Shh and its downstream effectors Ptch1 and Gli1 exhibited a significant increase in expression upon BAF170 overexpression. This effect was attenuated upon SIRT6 knockdown (Figs. 6i, j and S6F). SIRT6 has been reported to bond mono-ADP-ribosylate BAF170 on K312.^31^ We introduced BAF170 mutants and found that SCs with BAF170- K312A mutant failed to activate *Shh* transcription compared to that with BAF170- WT plasmids (*P* < 0.01, *n* = 5) (Figs. 6k, l and S6G).

Further, co-IP assay also revealed an interaction between endogenous c-Jun and BAF170 (Fig. 6m), which could be attributed to the intermediary role of SIRT6. To investigate whether c-Jun/SIRT6/BAF170 complex binds to injury-specific enhancers in SCs and cooperatively activates *shh* transcription, we reanalyzed H3K27ac and c-Jun ChIP-seq datasets (GSE190858 and GSE63103) in sham and injury SCs. As previously reported,^29^ three injury-activated and c-Jun bound enhancers sites were identified by mm10 coordinates: chr5:28557965–28558769 (E1); chr5:28567282–28568085 (E2); chr5:28630064–28630888 (E3). These were defined by prominent overlap between H3K27ac and c-Jun in injured SCs (Fig. S6C). To confirm the c-Jun/SIRT6/BAF170 complex binds to these enhancers, we performed ChIP-qPCR in SCs, and showed c-Jun and SIRT6 binding at E1 (*P* < 0.01, *n* = 3) and E3 (*P* < 0.05, *n* = 3) but not E2 (*P* > 0.05, *n* = 3). Moreover, BAF170 ChIP-qPCR in SIRT6-deficient SCs also revealed a declined enrichment in E1 (*P* < 0.05, *n* = 3) (Fig. 6n). Dual luciferase assay in HEK293T cells further confirmed that c-Jun activation of E1 and E3 can be modulated by SIRT6 and BAF170 (Figs. 6o and S6D). In conclusion, these results reveal that c-Jun/SIRT6/BAF170 complex activates *shh* transcription in SCs by binding to injury-specific enhancers (Fig. 6p).

### Conditional SIRT6 ablation in Plp1-lineage cells impairs GMT and subsequent bone repair

**Fig. 7:**
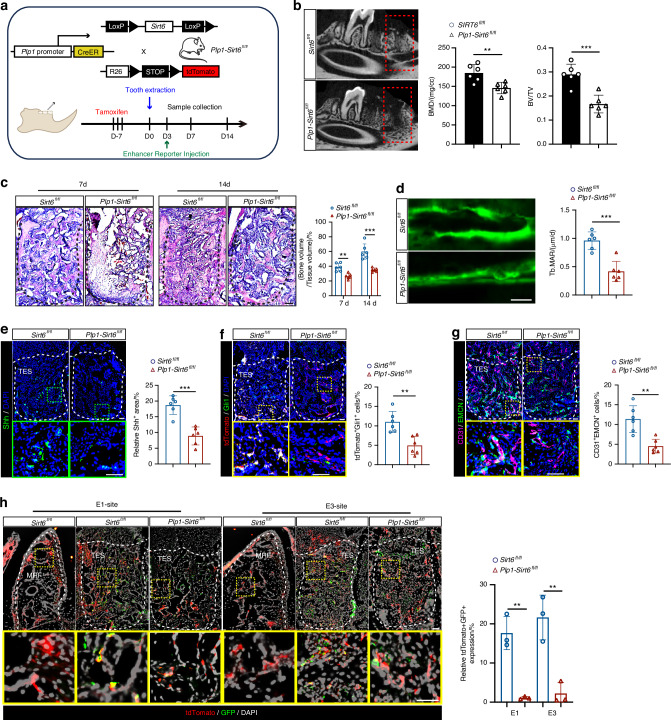
Conditional SIRT6 ablation in SCs impairs GMT and subsequent bone repair. **a** Experimental scheme for conditional SIRT6 deletion in SCs from jaw bone healing mice model. **b** Representative images of μCT reconstruction of the alveolar bone regeneration at day 7 post tooth extraction in *Sirt6*^*fl/fl*^ and *Plp1-Sirt6*^*fl/fl*^ mice. The quantitative analysis of BMD and BV/TV was shown (*n* = 6). **c** *H&E* staining of TES at day 7 and day 14 post tooth extraction (*n* = 6). Scale bar: 100 μm. **d** Dynamic histomorphometry of trabecular bone (Tb) with quantification of MAR in TES (*n* = 6). Scale bar: 5 μm. **e** Representative images of Shh immunostaining in healing sockets at day 7 post tooth extraction and the relative quantification (*n* = 6). Scale bar: 100 μm. **f** Representative images of tdTomato^+^ cells and Gli1 immunostaining in TES at day 7 post tooth extraction and relative quantification per socket (*n* = 6). Scale bar: 100 μm. **g** Representative images of CD31 and EMCN immunostaining and quantification of CD31^+^EMCN^+^ type H vessels in TES (*n* = 6). Scale bar: 100 μm. **h** Reporter vectors (*Shh* E1 and E3-site-HSVtk promoter-GFP) were transfected into tooth extraction sockets by local delivery. Representative images of tdTomato^+^ SCs and GFP expression were shown and the percentage of tdTomato^+^GFP^+^ cells per socket was analyzed (*n* = 3). Scale bar: 100 μm. Data were presented as mean ± SD; **P < 0.01, ***P < 0.001

To assess the in vivo role of SIRT6 in the GMT of Plp1-lineage cells and subsequent bone repair, we employed a conditional SIRT6 knockout model in SCs using *Plp1-creER*^*T2*^*; tdTomato* mice. The experimental strategy involved tamoxifen administration and a tooth extraction model, as outlined in Fig. 7a. We observed a significant reduction in bone regeneration within TES of *Plp1-Sirt6*^*fl/fl*^ mice at both day 7 and day 14 post-extraction (*P* < 0.01, *n* = 6) (Figs. 7b and S7A, B). These mice also exhibited reduced bone volume and bone formation rate within the TES, indicative of delayed bone formation (*P* < 0.01, *n* = 6) (Fig. 7c, d). Further analyses revealed decreased expression of Shh (*P* < 0.001, *n* = 6) (Fig. 7e) and reduced numbers of tdTomato^+^Gli1^+^ cells (*P* < 0.01, *n* = 6) (Fig. 7f), with the total number of tdTomato^+^ cells and neural fibers unchanged (*P* > 0.05, *n* = 6) (Fig. S7C), underscoring an impaired GMT in Plp1-lineage cells from *Plp1-Sirt6*^*fl/fl*^ mice. In addition, these mice displayed fewer CD31^+^EMCN^+^ type H vessels (*P* < 0.01, *n* = 6) and diminished COL1-positive areas (*P* < 0.01, *n* = 6), highlighting compromised bone regeneration following the conditional deletion of SIRT6 in Plp1-lineage cells (Figs. 7g and S7D). These observations were corroborated by the failure to activate the expression of Shh, stromal markers (CD105 and Gli1), and the glial marker MAG in tdTomato^+^ cells from *Plp1-Sirt6*^*fl/fl*^ mice (Fig. S7F). To investigate the transcriptional control by SIRT6 at specific enhancers, we cloned the upstream regions of E1 and E3 enhancer sites ahead of a GFP reporter. These constructs were locally delivered to the TES three days post tooth extraction (Fig. 7a). GFP expression, which was strongly induced from both the E1 and E3 sites and was largely overlapped with tdTomato signals. The tdTomato^+^GFP^+^ co-expression was effectively abolished in TES from *Plp1-Sirt6*^*fl/fl*^ mice (*P* < 0.01, *n* = 3 for each group), indicating that transcriptional activation from these injury-induced enhancers is SIRT6-dependent (Fig. 7h).

### Hh signaling-driven GMT is involved in the healing of human mandibular injury

**Fig. 8:**
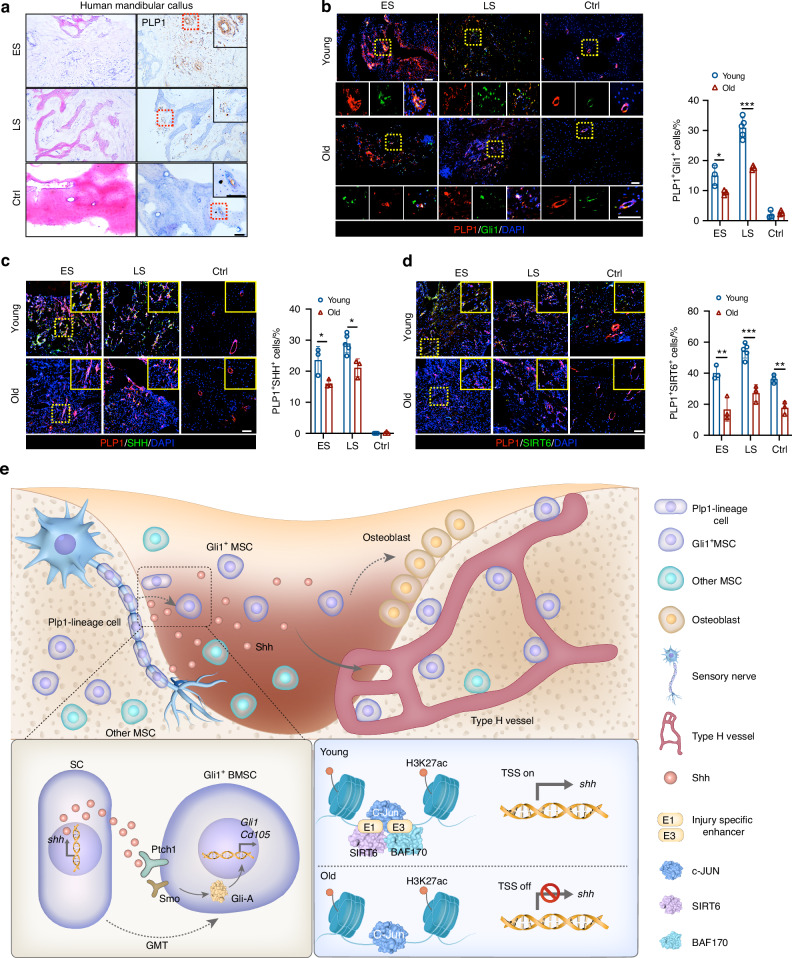
Hh signaling driven GMT process in human mandibular fracture callus from different stages and ages. **a** *H&E* staining and PLP1 immunohistochemical staining of clinical mandibular fracture callus from young patients. Scale bar: 100 μm. ES: Early-stage; LS: Late-stage; Ctrl: cortical bone specimens from uninjured mandible. **b** Representative images of PLP1 and Gli1 immunostaining in fracture callus from different stages and ages (*n* = 3 for the Young/Old-ES and Old-LS group, *n* = 5 for the Young-LS group). Scale bar: 100 μm. **c** Representative images of PLP1 and SHH immunostaining in fracture callus from patients with different stages and ages and the quantitative analysis (*n* = 3 for the Young/Old-ES and Old-LS group, *n* = 5 for the Young-LS group). Scale bar: 100 μm. **d** Representative images of PLP1 and SIRT6 immunostaining in fracture callus from patients with different stages and ages and the quantitative analysis (*n* = 3 for the Young/Old-ES and Old-LS group, *n* = 5 for the Young-LS group). Scale bar: 100 μm. **e** Schematic model: Shh signaling initiates the process of glia-to-mesenchyme transition of Plp1-lineage cells, contributing to the coupling of angiogenesis and osteogenesis and the alveolar bone regeneration. SIRT6-regulated injury-specific Shh enhancer is identified as a driver of this process. Data were presented as mean ± SD; *P < 0.05, **P < 0.01, ***P < 0.001

Finally, the Hh signaling-driven GMT in human mandibular fracture callus was further assessed. Both in the early (*n* = 3) and late (*n* = 5) stage of fracture callus, robust PLP1 immunoreactivity was observed in callus tissues and these PLP1^+^ cells were scattered within tissues adjacent to bone trabeculae (Fig. 8a). Specimens from uninjured mandible were used as the control group (*n* = 3) to demonstrate the minimal PLP1 immunoreactivity in quiescent state. Immunostaining of PLP1 and Gli1 in fracture callus from different stages and ages was performed. The number of PLP1^+^Gli1^+^ cells was markedly reduced in the old group compared to the young group both in the early (*P* < 0.05, *n* = 3) and late stage (*P* < 0.001, *n* = 5 for the Young group, *n* = 3 for the Old group) (Fig. 8b), suggesting an impaired GMT in human mandibular injury with aging. As expected, the SHH and SIRT6 expression in PLP1^+^ SCs also declined with aging in mandibular fracture callus (*P* < 0.05, *n* = 3 for the Young/Old-ES and Old-LS group, *n* = 5 for the Young-LS group) (Fig. 8c, d). Collectively, human fracture callus mirrors key elements of our experimental findings, including Hh signaling-driven GMT in the healing of bone injury. The schematic diagram of this study is presented in Fig. 8e.

## Discussion

Gli1^+^ stromal cells located around the NVB in craniofacial bones play pivotal roles in maintaining tissue homeostasis and facilitating regeneration.^24,36,37^ They are known to be heterogeneous with subsets contributing to skeletogenesis, chondrogenesis, or support of angiogenesis.^38,39^ However, the origin of such cells and how they respond to the injury had remained incompletely understood. The regeneration mode of in vivo dedifferentiation or transdifferentiation promised a stem cell-independent paradigm for tissue repair.^40^ Escalating studies have supported the existence of in vivo reprogramming in injured or tumor tissues.^41–43^ Notably, as neural crest-derived cells, SCs or Schwann cell precursors have been proved to directly contribute to neurons, odontoblasts, and skeletal progenitors in both developing embryo and injured sites.^18,23,44,45^ Repair SCs seem to manifest characteristics reminiscent of their parental neural crest population.^46^ We demonstrated here that regionally activated SCs transdifferentiated into Gli1^+^ stromal cells driven by injury-induced Shh enhancement by means of enhancer-regulated transcriptional activation. Moreover, we revealed that this GMT process was controlled by SIRT6-regulated ADP-ribosylation activity and its deficiency enabled the pronouncedly impaired jaw bone regeneration during aging. These findings offer a refined conceptual framework for understanding the regeneration mechanisms of craniofacial bones and potentially other injured tissues, highlighting the critical roles of molecular signaling pathways and cellular plasticity in tissue repair and regeneration.

In mammals, the peripheral nervous system retains a remarkable capacity for regeneration following nerve injury,^47^ largely due to the substantial plasticity of SCs.^48^ After nerve transection, the proximal and distal termini of the nerve diverge, leading to the proliferative outgrowth of SCs from the severed end of the distal stump.^49^ Given that SCs accompany nerves throughout the body during development, they possess significant potential to facilitate the repair and regeneration of various adult tissues. Recent research has indicated that SCs, following alveolar bone injury, can promote bone regeneration through paracrine mechanisms. These secreted factors significantly enhance the proliferation of alveolar skeletal stem cells,^50^ highlighting the influential role of SCs in tissue repair. In our study, Plp1-lineage SCs demonstrated the potential to transdifferentiate into Gli1^+^ stromal cells. These cells, while negative for classical osteo-lineage progenitor cell markers,^51^ are capable of promoting bone regeneration by regulating the formation of specialized CD31^+^EMCN^+^ type H vessels.^11^ *Plp1-CreER*^*T2*^ transgenic mice were employed to trace SCs in this study. However, it is important to note that upon activation with tamoxifen, *Plp1-CreER*^*T2*^ targets not only SCs but also oligodendrocytes, as well as some non-neural cells in the heart and gonads.^52,53^ Additionally, *Sox10-cre; tdTomato* and *Gli1-creER*^*T2*^*; tdTomato* mice were employed to provide further evidence supporting the presence of glia-to-mesenchyme transition during bone repair. During our scRNA-seq analysis, we did not identify independent SCs population at single-cell resolution, possibly due to methodological challenges. However, we did identify a unique subcluster of Gli1^+^ cells co-expressing neural glial and stromal markers, with enrichment in the Shh signaling pathway. This suggests an intermediate stage in the transition from SCs to MSCs, indicating the GMT process (Fig. 2). Further lineage tracing experiments also supported the GMT process during the healing of jaw bones (Fig. 3).

SCs undergo a notable reprogramming process after injury, characterized by the significant downregulation of myelination-associated proteins and the upregulation of repair markers that facilitate regeneration.^25,35^ Among these, Shh stands out as a unique marker of the SC repair state, transitioning from no expression in neural crest-derived mature SCs to becoming a pivotal player in injury response.^54^ The c-Jun subunit of the pioneer transcription factor AP-1 is known to bind to a series of injury-induced enhancers upstream of the *Shh* gene in SCs,^25^ highlighting its role in the activation of repair mechanisms. Our data further link the GMT process to age-related changes, pinpointing the longevity-associated gene SIRT6 as a cooperative partner of c-Jun through its ADP-ribosylation activity. Mechanistically, the c-Jun/SIRT6/BAF170 complex directly binds to injury-specific enhancers to activate *Shh* transcription, enhancing downstream stromal cell marker expression through paracrine or autocrine signaling (Fig. 6).

To explore the in vivo role of this SIRT6-regulated mechanism in jaw bone regeneration, we utilized conditional SIRT6 knockout mice, revealing that SIRT6 deficiency led to impaired Plp1-lineage cells transition and delayed healing in TES. The identification of two injury-specific enhancers further validated their dependence on SIRT6, underscoring the importance of SIRT6-regulated Shh expression in preventing age-related jaw bone defects (Fig. 7h). Additionally, assessments of human mandibular fracture callus supported the critical role of SC transition and Hh signaling activation during the healing process.

In summary, our findings unveil the previously unappreciated potential of Plp1-lineage cells transition in facilitating the coupling between Gli1^+^ MSCs osteogenesis and revascularization during the healing of bone injury. Deciphering this process and its affiliated molecular mechanisms holds promise for advancing our understanding of its implications for development, regeneration, and disease in other tissues, potentially paving the way for novel therapeutic approaches.

## Materials and methods

### Human subjects

Mandibular fracture callus tissues (including the body of mandible and alveolar bone regions) used in this study were from male donors and were collected during maxillofacial surgery at the Department of Oral and Maxillofacial Surgery, Affiliated Hospital of Stomatology, Nanjing Medical University. These patients had their cranial and cerebral injuries ruled out before being referred to our institution. Consequently, many of these cases presented with malunion, necessitating surgical intervention to reposition and stabilize the bones properly. This approach was essential to restore both the functional and esthetic aspects of the jaw. The control (uninjured) mandible samples were collected from surplus bones of donors removed undergoing orthognathic surgery at our hospital. Patients ranged from 18 to 30 years were classified as young group, while patients with the age over 60 were classified as old group. The fracture diagnosis was confirmed on an initial radiograph in all patients. Different stages of mandibular fracture healing and callus formation were defined according to previous study (stage 1–2: early stage; stage 3–6: late stage).^55^ All patients provided written informed consent for the study. The Ethics Committee of Nanjing Medical University approved the study protocol. (approval number: [2023]-395).

### Mice

The Ethics Committee of Nanjing Medical University sanctioned all animal experiment protocols, which adhered to the institution’s Animal Care Committee guidelines (approval number: IACUC-2311043). The following strains were obtained from The Jackson Laboratory: *Plp1-creER*^*T2*^ (Stock No. #005975), *Rosa26-DTA* (Stock No. #009669), and *Sirt6*^*fl/fl*^ (Stock No. #017334). *Rosa26-tdTomato* (Stock No. #T002249) mice were purchased from GemPharmatech (Nanjing, China), *Sox10-2A-cre* (Stock No. #NM-KI-220066) and *Gli1-creER*^*T2*^ (Stock No. #NM-KI-200073) were purchased from Shanghai Model Organisms Center, Inc. Cre-recombinase activation was induced through intraperitoneal injections of 200 mg/kg tamoxifen (Sigma) for five consecutive days. The male mice used in further experiments were maintained in a specific pathogen-free facility and were subjected to controlled temperature conditions and a 12-h light/dark cycle.

### Inferior alveolar denervation mouse model and alveolar injury model

Male mice were administered anesthesia via an intraperitoneal injection of a ketamine/xylazine mixture at a dosage of 0.1 mL per 20 g of body weight, comprising 87.5 mg/kg Ketamine and 12.5 mg/kg Xylazine. For IAN denervation, one-side IANs were severed using microsurgery as previously described.^21^ Briefly, a carefully contoured incision was made, extending from the angle of the right mandible to the posterior region of the neck. With meticulous care to avoid damaging any vascular structures, the posterior section of the hemimandible was skillfully retracted laterally from the base of the skull to unveil the IAN. This nerve was then transected before it could enter the mandibular canal. In contrast, sham surgeries involved the same dissection and retraction steps but preserved the integrity of the IAN. A postoperative period of at least 2 weeks was allotted to ensure complete nerve degeneration following denervation, before proceeding with any subsequent experiments. The alveolar injury model was established via tooth extraction. Briefly, the right mandibular first molars were extracted using a 26G syringe needle and forceps, aided by a stereomicroscope. Sterile gauze was placed in the socket for hemostasis. The mice were placed on a temperature-regulated heating pad and given a soft diet after surgery and no accidental death happened.

### von Frey testing

Behavioral testing for von Frey filament stimulus and head withdrawal was performed. Before behavioral testing, mice received daily training in which they were guided into a dark plastic cage with their snouts and mouths protruded through a hole in the side of the cage. Mice acclimated to their environment for a minimum of 15 min. Using ascending mechanical intensities, von Frey filaments were used in ascending mechanical intensities to stimulate the right facial skin above the mental foramen. A positive response was noted if the mouse withdrew its head during filament application or immediately after removal. The 50% response threshold was subsequently determined based on multiple stimulations.

### Chemical injection and in vivo reporter assay

Vismodegib (GDC-0449, MCE) was administered intraperitoneally at a dosage of 1.5 mg per 30 g body weight bi-daily, with a 6-h interval for indicated days. *Shh Enhancer1*-site and *Shh Enhancer3*-site were cloned into the front of a GFP reporter vector (purchased from Shanghai Genechem Co., Ltd.). Plasmids of *Shh Enhancer1*-site and *Enhancer3*-site-HSVtk promoter-GFP were efficiently transfected into the mesial and distal mucosa of the buccal and palatal sides of mandibular TES using a microsyringe.

### Micro-computed tomography (micro-CT) analysis

The collected mandibles were fixed in 4% paraformaldehyde (PFA) at 4 °C for 1 d and then stored in 0.5% PFA at 4 °C before being scanned. The samples were scanned with a micro-CT system (Skyscan 1176, Belgium) at a high resolution (15 μm) with an energy of 50 kV and 456 μA. The region of interest (VOI) was limited to the anatomical root area. Lamina dura could be regarded as a radiographic landmark after tooth extraction. The VOI differences between lamina dura and new formed bone were applied to define “tooth socket”. NRecon v1.6 and CTAn v1.13.8.1 software were used for data analysis and image processing.

### Histology, Immunofluorescence, and fluorescence labeling analysis

Tissue samples were fixed in 4% PFA at 4 °C overnight, followed by washing in phosphate-buffered saline (PBS). Subsequently, the specimens underwent decalcification in a 15% EDTA solution, dissolved in PBS, at 4 °C for a duration of 4 weeks, with the EDTA solution being refreshed every 48 h. Post-decalcification, the tissues were dehydrated, embedded in paraffin, and sectioned to a thickness of 6 μm. Hematoxylin and eosin staining were conducted in compliance with the manufacturer’s protocol (Biosharp).

For immunofluorescence analyses, the decalcified samples were submerged in a 30% sucrose solution, followed by embedding in optimal cutting temperature compound. They were then sectioned to 15 μm thickness using a cryostat microtome (Leica). These sections underwent a PBS wash and were permeabilized using 0.5% Triton X-100 (Sigma). Following permeabilization, the specimens were blocked and subsequently incubated with the primary antibody at 4 °C overnight. The antibodies used in immunofluorescence are listed in Table S1. After incubation with primary antibodies, slices were washed with PBS and incubated with FITC, Cy5, or Cy3-labeled secondary IgG at 37 °C for 1 h. Finally, sections were labeled with DAPI and imaged by fluorescence microscope (Leica Microsystems, Germany).

For fluorescence analysis, 7 days after tooth extraction, mice received a subcutaneous calcein injection (10 mg/kg; Sigma, USA) 7 and 2 days prior to euthanasia. Mandibles underwent graded sucrose dehydration (15%–30%) before embedding in methyl methacrylate. Specimens were sectioned at 20 μm using a freezing microtome (EXAKT, Germany). The calcein band gap was visualized with a fluorescence microscope (Leica Microsystems, Germany) and quantified using ImageJ software.

### Single-cell dissociation

Three Sham or Denervation 12-week-old male C57BL/6J wild-type mice were combined to extract single-cell suspensions. Three days after all three molars tooth extraction, mandibles were meticulously isolated under a stereomicroscope. Soft tissues and jaw bone around the incisor and behind condyle were cut off to obtain the region of TES tissues. These samples were rinsed with PBS and then finely minced (~1 mm^3^) on ice. The tissues were subjected to enzymatic digestion using a combination of 2 mg/mL collagenase I (Biosharp) and 2 mg/mL collagenase II (Biosharp) for 45 min at 37 °C under gentle agitation. Post-digestion, the resultant mixture was filtered through a 70 μm cell strainer, followed by centrifugation at 300 *g* for 5 min. Discarding the supernatant, the cell pellet was resuspended in red blood cell lysis buffer (Miltenyi Biotec) to eliminate erythrocytes. Following a wash step in PBS supplemented with 0.04% BSA, the cell pellets were redissolved in the same buffer and subsequently sieved through a 40 μm cell strainer. The dissociated single cells underwent viability staining with Calcein-AM and Draq7. This single-cell suspension was further refined using the MACS Dead Cell Removal Kit (Miltenyi Biotec).

### Single-cell RNA sequencing

The BD Rhapsody system was employed for high-resolution single-cell transcriptomic profiling. A methodological strategy involving limited dilution was utilized to stochastically distribute the single-cell suspension across over 200 000 microwells. To ensure optimal interactions, oligonucleotide-barcode-carrying beads were introduced to saturation, facilitating a one-to-one association of each bead with a cell within a microwell. Within these microwells, cellular lysis was induced, allowing mRNA molecules to hybridize with the barcoded capture oligonucleotides present on the beads. Subsequently, these beads were consolidated into a single reaction vessel for reverse transcription and ExoI digestion processes. It is noteworthy that, during the cDNA synthesis phase, each cDNA fragment was uniquely labeled on its 5′ end (corresponding to the 3′ end of an mRNA transcript) with a UMI and a cell-specific barcode, denoting its cellular origin. For comprehensive transcriptomic analysis, the BD Rhapsody’s single-cell whole-transcriptome amplification (WTA) protocol was adopted. This encompassed random priming and extension (RPE), RPE-targeted PCR amplification, and WTA index PCR. For accurate library quantification, tools such as the High Sensitivity DNA chip on the Bioanalyzer 2200 and the Qubit High Sensitivity DNA assay (Thermo Fisher Scientific) were utilized. The resulting libraries were subsequently sequenced using an Illumina sequencer, based in San Diego, CA, under a 150 bp paired-end run configuration.

### scRNA-seq data processing

We employed the BD Rhapsody Analysis pipeline (Version 1.8, accessible at https://hub.docker.com/r/bdgenomics/rhapsody) using its default settings for read alignment and feature-barcode matrix generation. Only cells exhibiting between 200 and 6 000 expressed genes, and a mitochondrial UMI rate of less than 20%, were retained post cell-quality filtration. Moreover, mitochondrial genes were subsequently excluded from the expression matrix. For normalization and regression based on this expression matrix, the Seurat package was invoked, taking into account the UMI counts per sample and the respective mitochondrial rate, resulting in the generation of scaled data. A principal component analysis (PCA) was performed on the scaled data, focusing on the top 2 000 highly variable genes. The leading 20 principal components were then harnessed for UMAP projection. Through the application of a graph-based clustering approach, we derived unsupervised cell clustering results based on the top 10 principal components from the PCA. Subsequently, marker genes were ascertained using the ‘FindAllMarkers’ function.

### Pseudotime analysis

We conducted a Single-Cell Trajectory analysis using Monocle 2. Prior to the Monocle analysis, we selected marker genes derived from the Seurat clustering outcomes, as well as the raw expression counts of cells that met the quality criteria. Following this, pseudo-time analysis was employed, upon which the Branch Expression Analysis Modeling was utilized to ascertain genes determining branch fate.

### Flow cytometry

To define and determine the frequencies of TESs cells from *Plp1-creERT2; tdTomato* mice, collagenased mandibular TES cell suspensions were obtained and stained with the following antibodies: anti-CD45.2 (1:100), anti-CD144 (1:200), anti-Ter119 (1:25), anti-CD51 (1:50), anti-Sca1 (1:50) all from Biolegend, and anti-CD31 (1:100) from BD Biosciences. Alexa Fluor 488-conjugated Streptavidin (1:100, Invitrogen) was employed for the detection of cells targeted with the biotinylated anti-CD51 antibody. FlowJo v.10 software was used for the data analysis.

### Fluorescence-activated cell sorting (FACS) and cell culture

Collagenased mandibular TES fraction cell suspensions from *Plp1-creERT2; tdTomato* mice were obtained and tdTomato^+^ cells were sorted by MoFlo Astrios cell sorter Instrument (Beckman, USA). FACS-sorted cells were used for protein or RNA isolation and the osteogenic differentiation assay. For the isolation of SCs from sciatic nerves, bilateral segments of the sciatic nerve were harvested from wild-type C57BL/6J mice. Post-excision, the epineurium and fibrous tissues were meticulously dissected using fine forceps under a stereomicroscope. Subsequently, the tissues underwent incubation with an enzymatic cocktail comprising 0.25% Dispase II and 0.05% Collagenase I at 37 °C for 1 h, optimizing the digestion process to facilitate the maximal release of SCs. Finally, the products resulting from enzymatic digestion were subjected to filtration, followed by collection through centrifugation.

For cell culture, primary Schwann cells collected both from FACS or sciatic nerves were seeded onto tissue culture dishes coated with laminin. They were cultured in DMEM/F12 medium supplemented with 20% N2, 10% FBS, 1% P/S, and 10 μmol/L forskolin. Cells were passaged once they reached ~80%–90% confluency. Schwann cells from either passage 1 or 2 were utilized.

### Western blot and co-immunoprecipitation

Cells were harvested and subsequently lysed in RIPA buffer (Cat# P0013B, Beyotime) supplemented with a 10 mmol/L protease inhibitor. This mixture was kept on ice for 30 min and proteins from the lysate were separated using 10%–15% SDS-PAGE gels. And then the proteins were transferred onto Immobilon-PVDF membranes (Millipore, USA). To block non-specific binding, the membranes were treated with 5% fat-free milk at room temperature for 2 h and subsequently incubated with primary antibodies at 4 °C overnight. Detailed information about the primary antibodies is listed in Table S1. After washing with TBST three times, the membranes were further incubated with secondary antibodies conjugated with peroxidase for 1 h. Finally, the protein bands were visualized using an ECL detection kit (Millipore, USA).

For the co-IP assay, cellular lysis was achieved by treating the cells with IP lysate buffer (Beyotime Biotechnology, China), which was further supplemented with 1% Halt Protease & Phosphatase Inhibitor Cocktail (Thermo, USA). This lysis process was conducted on ice for a duration of 30 min, after which the cell lysate was obtained through centrifugation. The resulting supernatant was carefully collected and subsequently transferred into new Eppendorf tubes, where it underwent immunoprecipitation. The supernatant was incubated with a specific antibody and combined with 50 μL of protein A/G agarose beads (Roche, Germany) at 4 °C overnight. Following the overnight incubation, the mixture was subjected to three rounds of washing using 1× washing buffer. Subsequently, the beads were resuspended in 1× SDS loading buffer, boiled for 10 min, and prepared for Western blot analysis. A detailed list of the antibodies employed in this study can be found in Table S1.

### RT-qPCR and ChIP-qPCR assays

Total RNA was extracted from cells using Trizol reagent and reverse transcription was performed with the PrimeScript RT Reagent Kit (Takara Bio, Japan). The levels of each mRNA were normalized to the *Gapdh* level. Each experiment was conducted in triplicate. Expression of genes of interest was quantified using 2^−ΔΔCT^ method and the primer sequences used in this study are listed in Table S2.

The ChIP-qPCR assay was performed in accordance with the manufacturer’s instructions, utilizing the SimpleChIP Plus Sonication Chromatin IP Kit (Cell Signaling Technology, USA). In brief, SCs were harvested, crosslinked, and subsequently lysed. Chromatin pellets were sonicated in ChIP dilution buffer to generate DNA fragments. The resultant supernatant was further diluted with ChIP buffer and subjected to an overnight incubation at 4 °C with constant rotation in the presence of primary antibodies. Detailed information regarding the antibodies can be found in Table S1. Protein G magnetic beads were then introduced to each sample, followed by a 2-h incubation at 4 °C with rotation. Chromatin was eluted using ChIP Elution Buffer, and cross-links were reversed at 65 °C for a duration of 2 h. Ultimately, DNA was extracted and purified for subsequent qPCR analysis. Primer sequences for the three *Shh* enhancers are provided in Table S4.

### Transfection and luciferase reporter assay

All siRNAs were obtained from GenePharma (Shanghai, China) and the sequences are provided in Table S3. A mouse SIRT6 overexpression plasmid, SIRT6 separation of function mutant expression plasmids (R65A, H133Y, G60A), and BAF170 separation of function mutant expression plasmid (K312A) were synthesized by Hanyinbt (Shanghai, China). The mouse/human c-JUN overexpression plasmids were from GeneCopoeia. SCs or 293T cells were transfected using LipoRNAi (Beyotime Biotechnology, China) according to the manufacturer’s instructions.

For luciferase reporter assay, two mouse *Shh* enhancers (E1, E3) were extracted from mouse genomic DNA. These enhancers were positioned upstream of the pGL4 luciferase reporter with the E1B TATA promoter. HEK293T cells were transfected with these enhancers, human c-JUN overexpression plasmid, and si-SIRT6/si-BAF170 via LipoRNAi. After 48 h, cells were harvested for the dual-luciferase assay using Dual-Luciferase Reporter Assay System (Promega, USA). For each sample, Firefly luciferase activity was normalized to Renilla luciferase activity.

### Fluorescence in situ hybridization (FISH) assay

FISH probes were directly labeled with Fluorescent In Situ Hybridization Kit (Genepharma, Shanghai, China) according to the manufacturer’s instructions. The probes were designed and synthesized by Genepharma (Shanghai, China). Sections were added with Proteinase K and incubated at 37 °C for 20 min. Then, each tissue section was washed with 2× SSC wash buffer at room temperature for three times and rehydrated through an alcohol gradient of 2 min each (70%, 80%, 90%, and 100% alcohol). Next, 100 μL of prewarmed denaturation solution was applied to the sample and incubated for 8 min at 78 °C and then the section was rehydrated through an alcohol gradient again. After that, the tissue was incubated with the *Shh* FISH probe (5′-AATCGTTCGGAGTTTCTTGTGACAGGTGCCAATGTGGTAGAGCATGTCCACTGCTCGACCCTC-3′) at 37 °C for 12 h. These sections were washed with a prewarmed 2× SSC wash buffer for 15 min. After washing with PBS for 10 min, the sections were observed by fluorescence microscope (Leica Microsystems, Germany).

### NAD^+^ measurement

For NAD^+^ measurements, young/old SCs were resuspended and lysed with 200 μL NAD^+^/NADH extract buffer and centrifuged at 12 000 × *g* for 10 min on ice. The supernatant was collected to measure NAD^+^ using NAD^+^/NADH assay kit (Beyotime, China). NAD^+^ value was determined by calculating the value of total NAD^+^ and NADH (NAD^+^ = total NAD^+^−NADH).

### Osteogenic differentiation assay

For the induction of osteogenic differentiation, an initial cell seeding of 5 × 10^4^ cells purified from mandible of *Plp1-creER*^*T2*^*; tdTomato* mice by FACS were performed in 48-well plates. The cells were incubated in growth medium, either with Shh (at concentrations of 100 or 200 ng/mL) or a corresponding vehicle control, over a period of 5 days. Subsequently, the growth medium was replaced with osteogenic medium (comprising DMEM/F12 with 10% FBS) supplemented with dexamethasone (at a concentration of 10^−8^ mol/L), ascorbic acid (at 0.2 mmol/L), and β-glycerolphosphate (at 10 mmol/L) for a total of 21 days, with medium refreshment occurring every 3 days. Following this incubation period, the cells were washed twice with PBS, fixed with 3.7% formaldehyde for 5 min, and subjected to both Alizarin Red staining. Precipitates obtained from three independent ARS assays were dissolved in 10% cetylpyridinium chloride, and their absorbance was measured at a wavelength of 545 nm.

### Statistical analysis

All presented results are expressed as the mean ± standard deviation (SD) and are derived from a minimum of three independent experiments. Throughout the analysis period, the identifying information for each sample was securely sealed to prevent bias, and every data point underwent a rigorous verification process, involving independent checks by two examiners. Statistical analyses were performed using GraphPad Prism 8 software. For comparisons between two groups, statistical significance was evaluated through a two-tailed unpaired Student’s *t*-test. When comparing differences among more than two groups, a one-way analysis of variance was applied, followed by Tukey’s post hoc test for pairwise comparisons. *P* values of less than 0.05 were considered significant.

## Supplementary information

Supplemental material

## Data Availability

All data needed to evaluate the conclusions in the paper are present in the paper and the Supplementary Materials.
