# Supplementary material for: Enhancer-driven Shh signaling promotes glia-to-mesenchyme transition during bone repair
Source: Bone Res. 2025 Jan 26;13:16. doi: 10.1038/s41413-024-00396-8 (PMC11770102; doi:10.1038/s41413-024-00396-8)
Supplement: Supplementary file 1 — Supplemental material [file 41413_2024_396_MOESM1_ESM.pdf]

Supplementary Materials

Enhancer-driven Shh signaling promotes glia-to- mesenchyme transition during bone repair

Xin Shen, Hang Zhang, Zesheng Song, Yangjiele Dong, Xiao Ge, Shenghao Jin, Songsong Guo, Ping Zhang, Yu Fu, Yuchi Zhu, Na Xiao, Dongmiao Wang, Jie Cheng, Rongyao Xu*, Hongbing Jiang*

*Corresponding author. Email: jhb@njmu.edu.cn

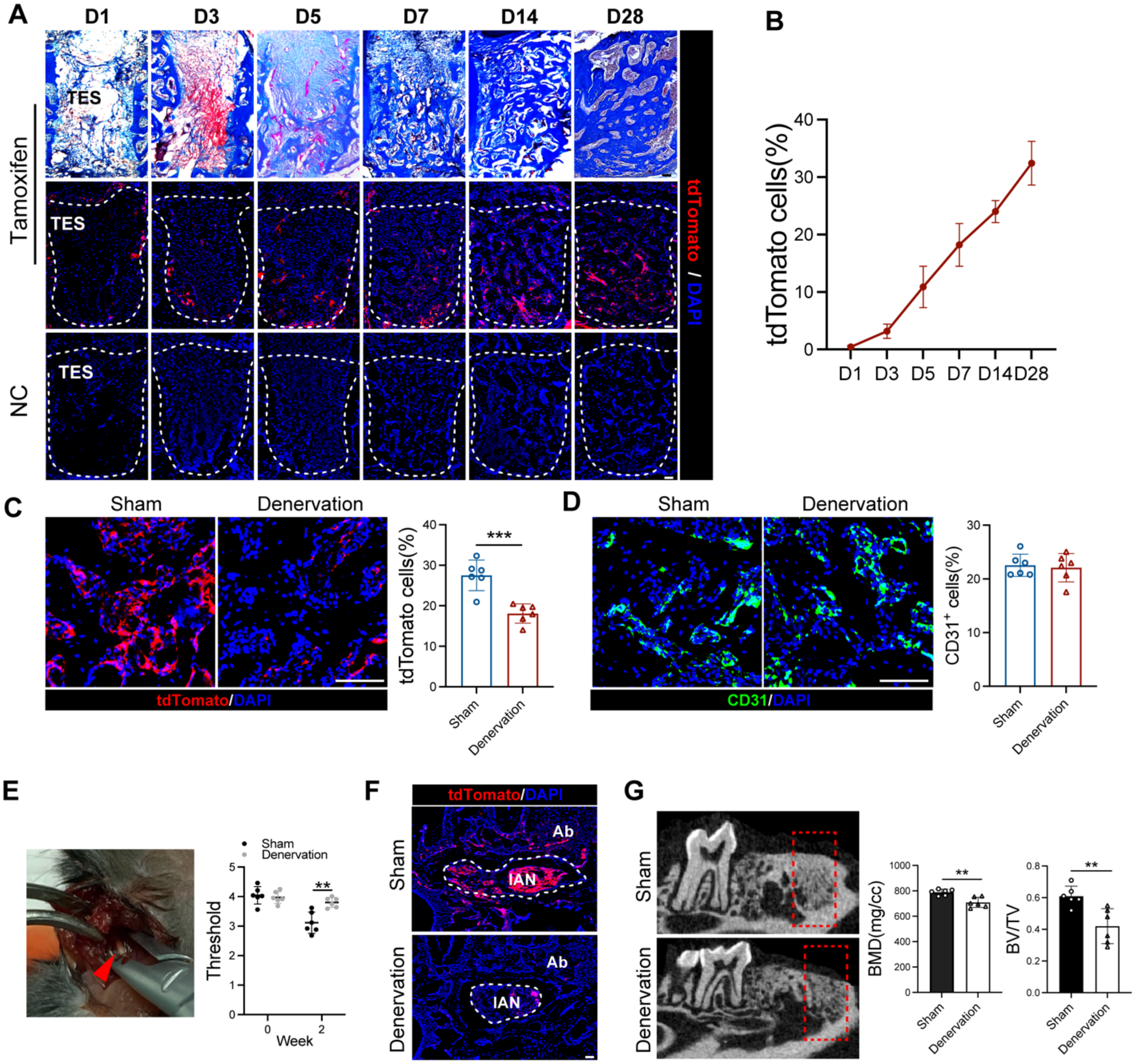

Figure S1. The construction of tooth extraction model and validation of hypoalgesia with surgical denervation, related to Figure 1.

(A) Representative images of masson trichrome staining and the tdTomato^{+} cells during healing process of tooth extraction sockets. Scale bar: 100 µm. NC: Negative Control group refer to *Plp1-creER^{T2}; tdTomato* mice without tamoxifen pre-treatment, TES: tooth extraction socket. (B) Quantification of tdTomato^{+} cells in TES of *Plp1-creER^{T2}; tdTomato* mice treated with tamoxifen. (n=3). (C) Representative images of tdTomato^{+} cells in alveolar bones from Sham and Denervation mice (n=6). Scale bar: 100 µm. (D) Representative images of CD31 immunostaining in alveolar bones from Sham and Denervation mice (n=6). Scale bar: 100 µm. (E) Inferior alveolar denervation surgical procedures. Red arrows: exposed inferior alveolar nerve. von Frey testing at

2 wk post-injury with or without IAN denervation in comparison to Sham control (n=6). (F) Representative images of tdTomato^{+} cells in IAN from Sham and Denervation mice. Scale bar: 100 μm. (G) Representative images of μCT reconstruction of the alveolar bone regeneration at day 14 post tooth extraction and quantitative analyses on BMD and BV/TV (n=6). Data were presented as mean ± SD; $**p < 0.01$, $***p < 0.001$.

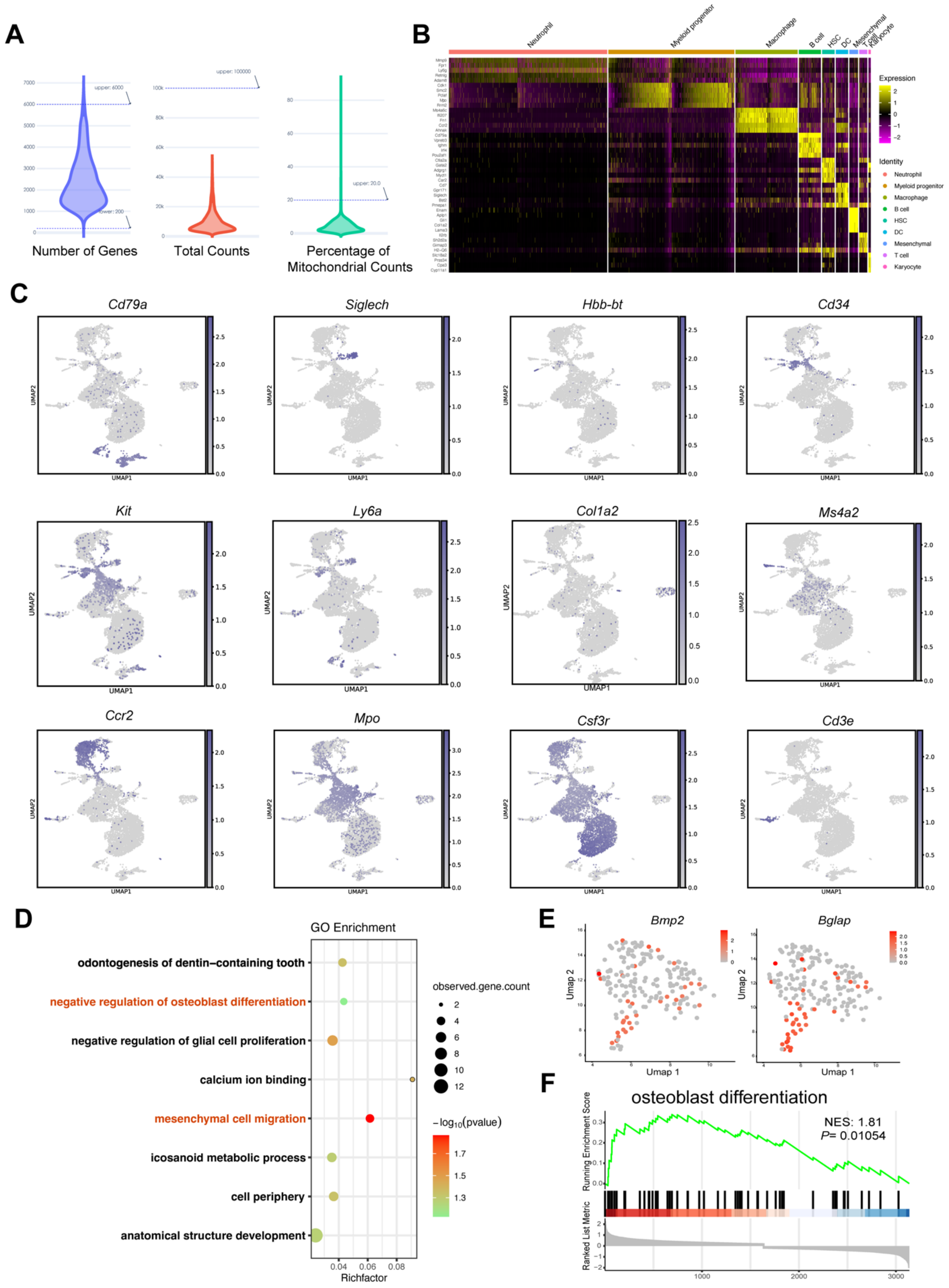

Figure S2. Additional description of cell identity by sc-RNA seq, related to Figure 2.

(A) The violin plots showed the Number of Genes, Total Counts and Percentage of Mitochondrial Counts. (B) Heatmap of 9 cell subtypes. After dividing the cells into 9 subtypes, top 5 genes with the highest expression in each subtype were identified and compared between the subtypes. (C) The expression levels of different marker genes were projected onto UMAP atlas. (D) GO enrichment analysis of cluster 2. (E) Cluster 3 express osteogenetic marker *Bmp2 and Bglap*. (F) GSEA analysis of osteoblast differentiation pathway in subcluster 3.

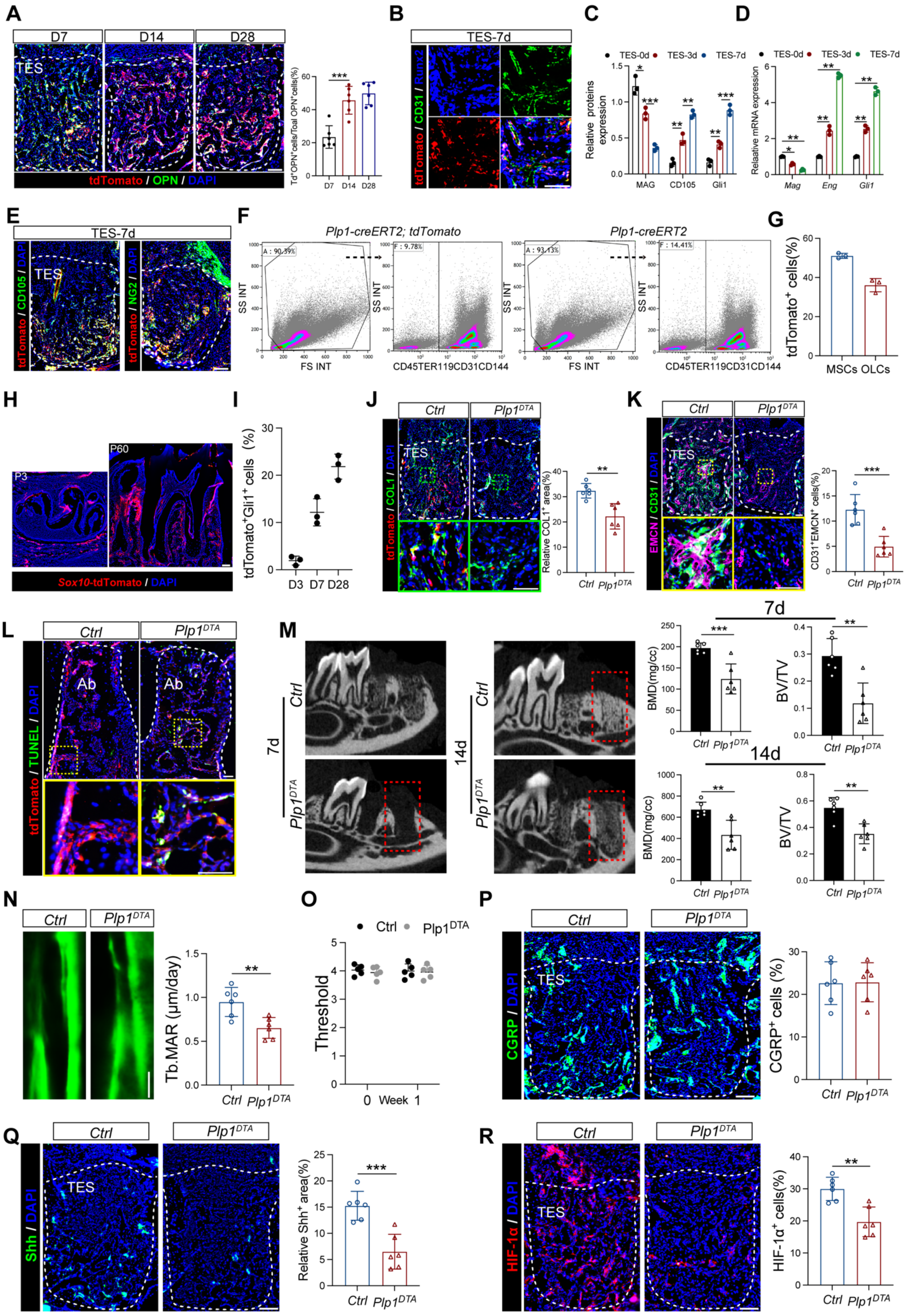

A
D7
D14
D28
TES
tdTomato / OPN / DAPI
Td+OPN+cells/Toal OPN+cells(%)
D7 D14 D28
B
TES-7d
tdTomato / CD31 / Runx2
C
TES-0d TES-3d TES-7d
Relative proteins expression
MAG CD105 Gli1
D
TES-0d TES-3d TES-7d
Relaative mRNA expression
Mag Eng Gli1
E
TES-7d
TES
tdTomato / CD105 / DAPI
tdTomato / NG2 / DAPI
F
Plp1-creERT2; tdTomato
Plp1-creERT2
A : 90.39%
F : 9.78%
A : 93.13%
F : 14.41%
SS INT
FS INT
CD45TER119CD31CD144
G
tdTomato+ cells(%)
MSCs OLCs
H
P3
P60
Sox10-tdTomato / DAPI
I
tdTomato+Gli1+ cells (%)
D3 D7 D28
J
Ctrl
Plp1DTA
TES
tdTomato / COL1 / DAPI
Relative COL1+ area(%)
K
Ctrl
Plp1DTA
TES
EMCN / CD31 / DAPI
CD31+EMCN+ cells(%)
L
Ctrl
Plp1DTA
Ab
tdTomato / TUNEL / DAPI
M
7d
14d
Ctrl
Plp1DTA
BMD(mg/cc)
BV/TV
N
Ctrl
Plp1DTA
Tb.MAR (μm/day)
O
Ctrl Plp1DTA
Threshold
0 Week 1
P
Ctrl
Plp1DTA
TES
CGRP / DAPI
CGRP+ cells (%)
Q
Ctrl
Plp1DTA
TES
Shh / DAPI
Relative Shh+ area(%)
R
Ctrl
Plp1DTA
TES
HIF-1α / DAPI
HIF-1α+ cells(%)

Figure S3. Plp1-lineage cells contribute to Gli1$^+$ MSCs formation and promote bone regeneration, related to Figure 3.

(A) Representative images of tdTomato$^+$ cells and OPN immunostaining during jaw bone regeneration and quantification of tdTomato$^+$ OPN$^+$ cells in TES from *Plp1-creER^{T2}; tdTomato* mice (n=6). Scale bar: 100 μm. (B) Representative images of tdTomato$^+$ cells and CD31, EMCN immunostaining in TES from *Plp1-creER^{T2}; tdTomato* mice. Scale bar: 100 μm. (C) The quantification of protein levels in Fig. 3C (n=3). (D) RT-qPCR data of *Mag*, *Eng* and *Gli1* mRNA expression of FACS-sorted tdTomato$^+$ cells in TES from *Plp1-creER^{T2}; tdTomato* mice (n=3). (E) Representative images of tdTomato$^+$ cells and CD105/NG2 immunostaining in TES from *Plp1-creER^{T2}; tdTomato* mice. Scale bar: 100 μm. (F) Supplementary gating strategies in Fig. 3E. (G) Quantitative data of flow cytometry plots in Fig. 3E (n=3). (H) Representative images of tdTomato$^+$ cells in mandible at postnatal day 3 (P3) and day 60 (P60). (I) The quantification of tdTomato$^+$ Gli1$^+$ cells in TES from *Sox10-cre; tdTomato* mice (n=3), related to Fig. 3G. (J) Representative images of tdTomato$^+$ cells and COL1 immunostaining in TES of control and *Plp1^{DTA}* mice at day 7 post tooth extraction and relative quantification per socket (n=6). Scale bar: 100 μm. (K) Representative images of tdTomato$^+$ cells combined with CD31 and EMCN immunostaining in TES of control and *Plp1^{DTA}* mice 7 days post tooth extraction and relative quantification per socket (n=6). Scale bar: 100 μm. (L) The TUNEL signal colocalizes with tdTomato$^+$ cells in *Plp1^{DTA}* but not control mice, confirming the efficacy and specificity of the cell ablation process. Scale bar: 100 μm. (M) Representative images of μCT reconstruction of the alveolar bone regeneration at day 7 and day 14 post tooth extraction and the quantitative analysis on BV/TV and BMD (n=6). (N) Dynamic histomorphometry of trabecular bone (Tb) with quantification of MAR in TES from *Plp1^{DTA}* and control mice (n=6). Scale bar: 5 μm. (O) von Frey testing at 1 week before and after tamoxifen administration in *Plp1^{DTA}* and control mice (n=5). (P-R) Representative images CGRP/Shh/HIF-1α immunostaining in TES of control and *Plp1^{DTA}* mice at day 7 post tooth extraction and relative quantification per socket (n=6). Scale bar: 100 μm. Data were presented as mean ± SD; *$p < 0.05$, ** $p < 0.01$, ***$p < 0.001$.

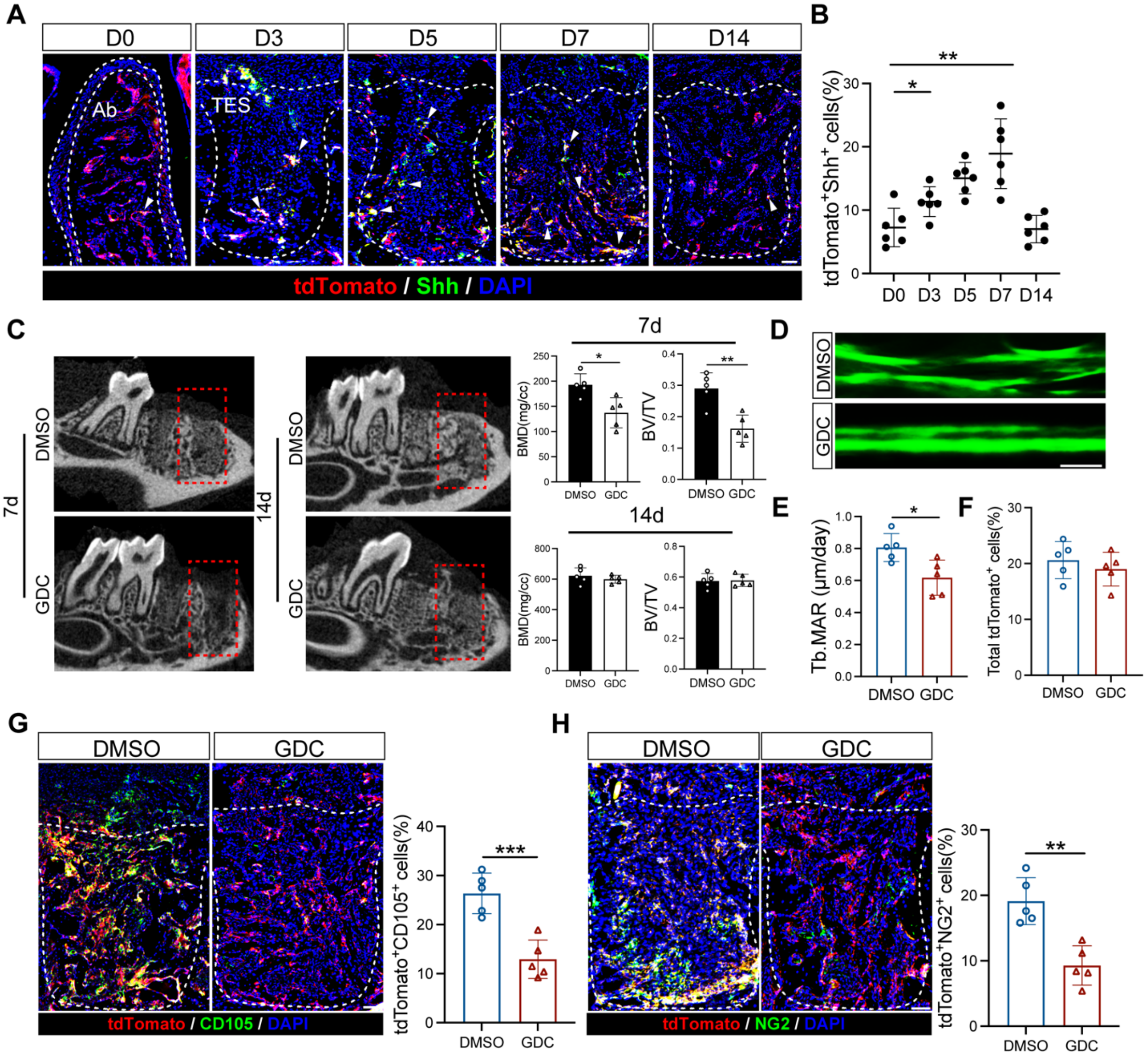

Figure S4. Shh driven Hh signaling promotes Plp1-lineage cells transition during bone regeneration, related to Figure 4.

(A and B) Representative images of tdTomato$^+$ cells combined with Shh immunostaining and the quantification of tdTomato$^+$ Shh$^+$ cells during the healing of jaw bone injury (n=6). Scale bar: 100 μm. (C) Representative images of μCT reconstruction of the jaw bone regeneration at day 7 and day 14 post tooth extraction and the quantitative analysis on BV/TV and BMD (n=5). (D and E) Dynamic histomorphometry of trabecular bone (Tb) with quantification of MAR in TES from DMSO and GDC mice (n=5). Scale bar: 5 μm. (F) The quatification of total tdTomato$^+$ cells in Fig. 4L (n=5). Scale bar: 100 μm. (G and H) Representative images of tdTomato$^+$ SCs combined with

CD105/NG2 immunostaining and the quantification in TES at day 7 post tooth extraction (n=6). Scale bar: 100 μm. Data were presented as mean ± SD; *$p < 0.05$, **$p < 0.01$, ***$p < 0.001$.

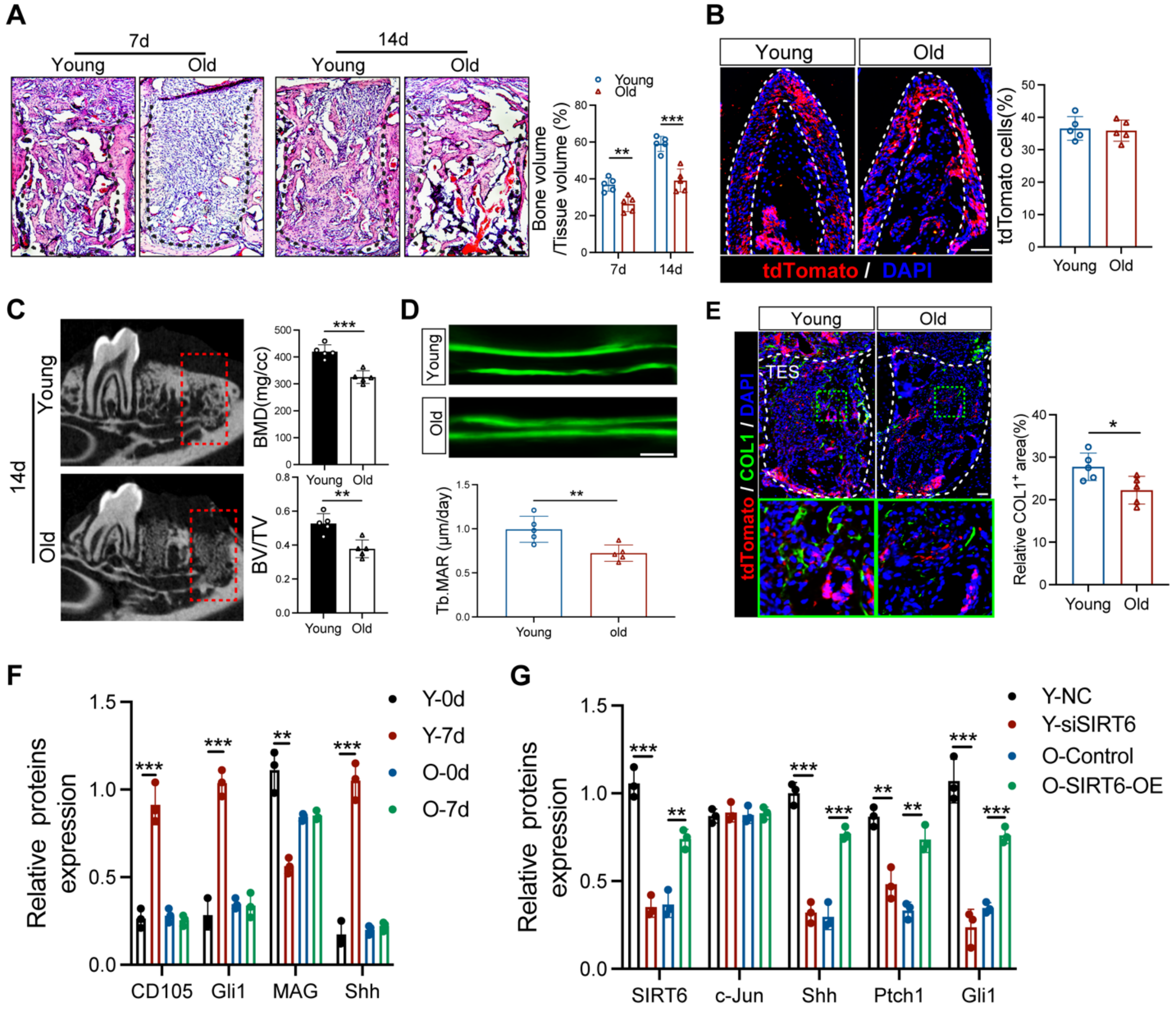

Figure S5. Impaired bone regeneration in aged mice, related to Figure 5.

(A) *H&E* staining of tooth sockets from young and old mice at day 7 and day 14 post tooth extraction (n=5). (B) Representative images of tdTomato^{+} cells in PDL and alveolar bone marrow before injury from young and old mice (n=5). Scale bar: 100 μm. (C) Representative images of μCT reconstruction of the alveolar bone regeneration at 14 days post tooth extraction from young/old mice and quantitative analyses (n=5). (D) Dynamic histomorphometry of trabecular bone (Tb) with quantification of MAR in TES from Young and Old mice (n=5). Scale bar: 5 μm. (E) Representative images of tdTomato^{+} cells and COL1 immunostaining in healing sockets at day 7 post tooth extraction and the relative quantification (n=5). Scale bar: 100 μm. (F) The

quantification of protein levels in Fig. 5F (n=3). (G) The quantification of protein levels in Fig. 5L (n=3). Data were presented as mean ± SD; *$p < 0.05$, **$p < 0.01$, ***$p < 0.001$.

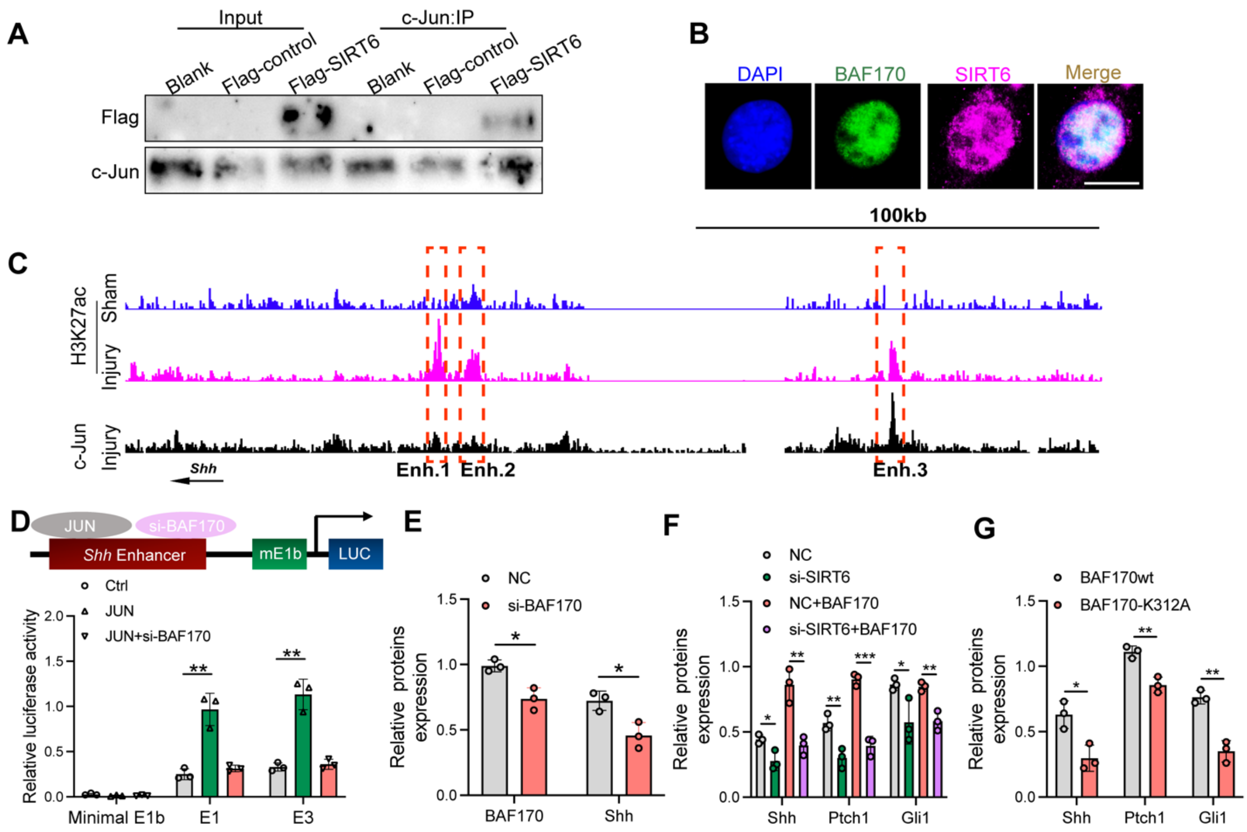

Figure S6. c-Jun/SIRT6/BAF170 cooperatively binds to injury-specific enhancers, related to Figure 6.

(A) Co-immunoprecipitation of c-Jun with ectopically expressed FLAG-tagged SIRT6 in 293T cells. (B) Immunofluorescence images of the colocalization of SIRT6 and BAF170 in SCs. Scale bar: 10 μm. (C) Analysis of H3K27ac and c-Jun ChIP-seq identified 3 injury-specific *shh* enhancers in SCs. (D) Luciferase reporter assay for shh enhancer 1 and 3 (n=3). (E) The quantification of protein levels in Fig. 6H (n=3). (F) The quantification of protein levels in Fig. 6J (n=3). (G) The quantification of protein levels in Fig. 6L (n=3). Data were presented as mean ± SD; **p* < 0.05, ***p* < 0.01, ****p* < 0.001.

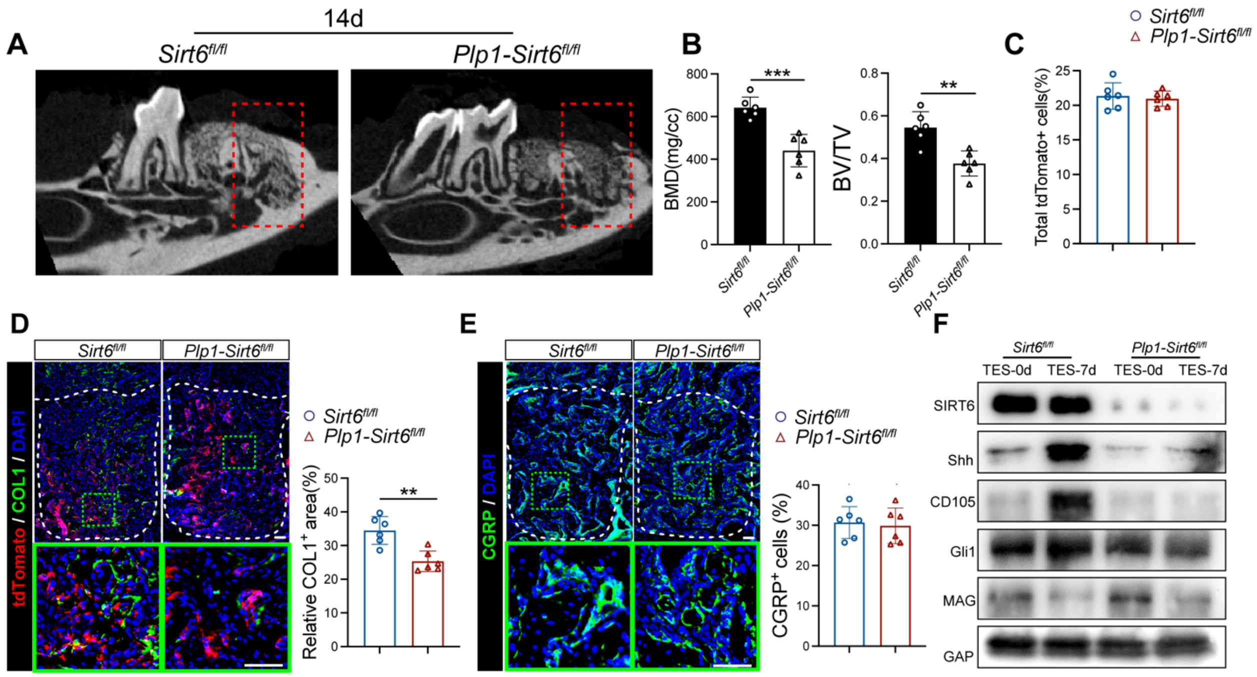

Fig. S7. Inhibited bone regeneration in *Plp1- Sirt6$^{fl/fl}$* mice and the Hh signaling driven GMT process in human mandibular fracture callus from different stages and ages.

(A) Representative images of µCT reconstruction of the jaw bone regeneration at day 14 post tooth extraction. (B) The quantitative analysis on BMD and BV/TV (n=6). (C) The quantitative analysis of total tdTomato^{+} cells in Fig. 7F (n=6). (D) Representative images of tdTomato^{+} cells and COL1 immunostaining in TES at 7 days post tooth extraction and relative quantification per socket (n=6). Scale bar: 100 µm. (E) Representative images of CGRP immunostaining in TES at 7 days post tooth extraction and relative quantification per socket (n=6). Scale bar: 100 µm. (F) Western blot images of SIRT6, Shh, CD105, Gli1 and MAG expression of FACS-sorted tdTomato^{+} cells from *Sirt6$^{fl/fl}$* and *Plp1- Sirt6$^{fl/fl}$* mice tooth extraction sockets at day 0 and 7 post tooth extraction. Data were presented as mean ± SD; **$p < 0.01$, ***$p < 0.001$.

Table S1. Reagent and resources

REAGENT and RESOURCE	SOURCE	IDENTIFIER
Antibodies		
Mouse monoclonal anti-CGRP	Abcam	Cat# ab81887
Rabbit polyclonal anti-COL1	Proteintech	Cat# 14695-1-AP
Rabbit polyclonal anti-CD31	Abcam	Cat# ab28364
Rat monoclonal anti-EMCN	Santa Cruz	Cat# sc-65495
Rabbit monoclonal anti-PLP	Abcam	Cat# ab254363
Rabbit monoclonal anti-Sox10	Abcam	Cat# Ab155279
Rabbit monoclonal anti-Gli1	Thermo Fisher Scientific	Cat# MA5-32553
Rabbit monoclonal anti-c-Jun	Cell Signaling Technilogy	Cat# 9165
Rabbit monoclonal anti- Shh	Abcam	Cat# ab53281
Mouse monoclonal anti-CD105	Abcam	Cat# ab230925
Rabbit monoclonal anti-MAG	Abcam	Cat# ab277535
Rabbit monoclonal anti-SIRT6	Cell Signaling Technilogy	Cat# 12486
Rabbit monoclonal anti-BAF170	Cell Signaling Technilogy	Cat# 12760
Mouse monoclonal anti-GAPDH	Abcam	Cat# ab8245
Mouse monoclonal anti-SIRT1	Abcam	Cat# ab110304
Rabbit monoclonal anti-SIRT2	Abcam	Cat# ab211033
Rabbit monoclonal anti-SIRT3	Abcam	Cat# ab246522
Rabbit polyclonal anti-PTCH1	Abcam	Cat# ab53715
APC anti-mouse CD45.2	Biolegend	Cat# 109814
APC anti-mouse CD144 (VE-cadherin)	Biolegend	Cat# 138011
APC anti-mouse CD31 (PECAM-1)	Biolegend	Cat# 160209
Pacific Blue (TM) anti-mouse Ly-6A/E (Sca-1)	Biolegend	Cat# 108210
Biotin anti-mouse CD51	Biolegend	Cat# 104103
Biological samples		
Mandibular fracture callus tissues	Department of Oral and Maxillofacial Surgery, Affiliated Hospital of Stomatology, Nanjing Medical University.	N/A
Chemicals, peptides, and recombinant proteins		
DMEM/F-12，HEPES	Thermo Fisher Scientific	Cat# 1133032
DMEM, high glucose	Thermo Fisher Scientific	Cat# 11965092
Fetal Bovine Serum	Thermo Fisher Scientific	Cat# 16140071

Penicillin-Streptomycin	Sigma-Aldrich	Cat# V900929
Calcein	Sigma-Aldrich	Cat# C0875
Alizarin Red S	Sigma-Aldrich	Cat# A5533
L-Ascorbic acid	Sigma-Aldrich	Cat# A5960
β-Glycerophosphate	Sigma-Aldrich	Cat# E2758
dexamethasone	Sigma-Aldrich	Cat# D4902
GDC-0449	MCE	Cat# HY-10440
Collagenase I	Biosharp	Cat# BS163
Collagenase II	Biosharp	Cat# BS164
Recombinant Mouse Shh protein	Abcam	Cat# ab276980
IP lysate buffer	Beyotime Biotechnology	Cat# P2177M
LipoRNAi™	Beyotime Biotechnology	Cat# C0535
TRIzol™ LS Reagent	Invitrogen	Cat# 10296010
Critical commercial assays		
Cell-Light EdU Apollo567 In Vitro Kit	RiboBio	Cat# C10310-1
Alkaline Phosphatase Assay Kit	Beyotime Biotechnology	Cat# P0321S
NAD^+/NADH Assay Kit with WST-8	Beyotime Biotechnology	Cat# S0175
Dual-Luciferase Reporter Assay System	Promega	Cat# E1910
SimpleChIP Plus Sonication Chromatin IP Kit	Cell Signaling Technilogy	Cat# 56383
Experimental models: Cell lines		
Primary Schwann cells	This paper	N/A
293T cells	ATCC	Cat# CM-1009
Oligonucleotides		
Primers for RT-qPCR and ChIP-qPCR see Table S1 and S3	Genepharma	N/A
Primers for siRNA see Table S2	Genepharma	N/A
Recombinant DNA		
Plasmid: pGL4.10- luciferase reporter vector	GeneCopoeia	N/A
Plasmid: PHY-810 (CMV-MCS-3XFlag-SV40-Neo) vector	Hanyinbt	N/A
E1-Site-pHSVtk-GFP	This paper	N/A
E3-Site-pHSVtk-GFP	This paper	N/A
Deposited data		
Single cell sequencing of TES from Sham and Denervation mice	This paper	N/A

ChIP-seq data for H3K27ac and c-Jun	GEO	GSE190858 and GSE63103
Software and algorithms		
Image J	Softonic	https://imagej.en.softonic.com/mac
LAS V4.12	Leica Microsystems	https://www.leicamicrosystems. com/products/microscope-software/
GraphPad Prism 8	GraphPad	https://www.graphpad.com/
Micro-CT NRecon v1.6 and CTAn v1.13.8.1	Skyscan	https://www.skyscan.pt/
FlowJo (v10)	Tree Star Inc	RRID: SCR_008520
Adobe Illustrator	Adobe	https://www.adobe.com/cn/products/illustrator.html
Seurat R package (v4.0.1)	Comprehensive R Archive Network	https://satijalab.org/seurat/
Monocle2 R package (v2.12.0)	Comprehensive R Archive Network	http://cole-trapnell-lab.github.io/ monocle-release/docs/
GSEA (v4.0.3)	N/A	http://www.gsea-msigdb.org/gsea/ index.jsp

Table S2. Primers for RT-qPCR

Genes	Farward	Reverse
Mag	GAGGGTTACGCCAGTTTGGA	GGTACAGGCTCTTGGCAACT
Eng	ATCACAGTGCTACCATCCCTTACC	CCAACTGGAGGACGATGCTTT
Gli1	CCTTTAGCAATGCCAGTGACC	GAGCGAGCTGGGATCTGTGTAG
Shh	CAATCTGCAACGGAAGCG	CAGGTGCCAATGTGGTAGAGC
Sirt1	CGCTGTGGCAGATTGTTATTAA	TTGATCTGAAGTCAGGAATCCC
Sirt2	CAGCTACTTCAAGAAACATCCG	TATTCTTTTCTGCAGGAGGTGT
Sirt3	TCTATACACAGAACATCGACGG	GCATGTAGCTGTTACAAAGGTC
Sirt4	GCATGTAGCTGTTACAAAGGTC	GTTGGTGAGAGGAGAATTGAGG
Sirt5	GTTGGTGAGAGGAGAATTGAGG	CATATTTGAACTTGGACGAGCC
Sirt6	CCCAAGTGTAAGACGCAGTA	CCCAAGTGTAAGACGCAGTA
Sirt7	CCAGGCACTTGGTTGTCTACAC	TAGGCTCCGCTTCGCTTAGGTC

Gapdh	GGTCGGTGTGAACGGATTTG	ATGAGCCCTTCCACAATG

Table S3. Primers for genes knockdown

si-SIRT1	CGGGAAUCCAAAGGAUAAUTT
si-SIRT2	GCACCUUCUACACAUCACATT
si-SIRT3	GGUGGAAGAAGGUCCAUAUTT
si-SIRT6	GCUACGUUGACGAGGUCAUTT
si-BAF170	GGACCCUCAACACCUUAUATT

Table S4. Primers for ChIP-qPCR

Genes	Farward	Reverse
Shh-Enhancer 1	TTGCACTTCTGCCTT TACCC	CAAACCTCGCCATGCTCTAT
Shh-Enhancer 2	GTGAAGTCAAGGGC CTGGTA	TCAGACCTTCCAAGCAGTCC
Shh-Enhancer 3	CAGTCCTATCTGGG CTGTCC	CCCCCAAACACAAAAACAAA
